# Transport of Drugs and Endogenous Compounds Mediated by Human OCT1: Studies in Single- and Double-Transfected Cell Models

**DOI:** 10.3389/fphar.2021.662535

**Published:** 2021-04-22

**Authors:** Bastian Haberkorn, Martin F. Fromm, Jörg König

**Affiliations:** Institute of Experimental and Clinical Pharmacology and Toxicology, Friedrich-Alexander-Universität Erlangen-Nürnberg, Erlangen, Germany

**Keywords:** HEK 293, double-transfected cell line, single-transfected cell line, P-glycoprotein, MATE1, OCT1, SLC22A1 (OCT1), MDCK cell line

## Abstract

Organic Cation Transporter 1 (OCT1, gene symbol: *SLC22A1*) is predominately expressed in human liver, localized in the basolateral membrane of hepatocytes and facilitates the uptake of endogenous compounds (e.g. serotonin, acetylcholine, thiamine), and widely prescribed drugs (e.g. metformin, fenoterol, morphine). Furthermore, exogenous compounds such as MPP^+^, ASP^+^ and Tetraethylammonium can be used as prototypic substrates to study the OCT1-mediated transport *in vitro*. Single-transfected cell lines recombinantly overexpressing OCT1 (e.g., HEK-OCT1) were established to study OCT1-mediated uptake and to evaluate transporter-mediated drug-drug interactions *in vitro*. Furthermore, double-transfected cell models simultaneously overexpressing basolaterally localized OCT1 together with an apically localized export protein have been established. Most of these cell models are based on polarized grown MDCK cells and can be used to analyze transcellular transport, mimicking the transport processes e.g. during the hepatobiliary elimination of drugs. Multidrug and toxin extrusion protein 1 (MATE1, gene symbol: *SLC47A1*) and the ATP-driven efflux pump P-glycoprotein (P-gp, gene symbol: *ABCB1*) are both expressed in the canalicular membrane of human hepatocytes and are described as transporters of organic cations. OCT1 and MATE1 have an overlapping substrate spectrum, indicating an important interplay of both transport proteins during the hepatobiliary elimination of drugs. Due to the important role of OCT1 for the transport of endogenous compounds and drugs, *in vitro* cell systems are important for the determination of the substrate spectrum of OCT1, the understanding of the molecular mechanisms of polarized transport, and the investigation of potential drug-drug interactions. Therefore, the aim of this review article is to summarize the current knowledge on cell systems recombinantly overexpressing human OCT1.

## Introduction

Transport proteins located in different membrane domains are important for the uptake, distribution and excretion of endogenous substances and drugs (International Transporter Consortium et al., 2010; König et al., 2013; Müller et al., 2018a; Koepsell, 2020). Whereas members of the SLC (Solute Carrier) transporter superfamily generally mediate the uptake of substances from the extracellular space into cells, members of the ABC (ATP-binding cassette) transporter superfamily are export proteins responsible for the energy-dependent export of substrates out of cells. SLC and ABC family members are important for the transport of a variety of approved drugs. Therefore, it is important to characterize drugs or drug metabolites as substrates or transport inhibitors. *In vitro* cell models are useful tools for this characterization. The importance of *in vitro* cell models is also highlighted by the fact that they are recommended as tools to study transporter-mediated drug interactions in the guideline/guidance of FDA Food and Drug Administration (2020) and EMA European Medicines Agency (2012).

**FIGURE 1:**
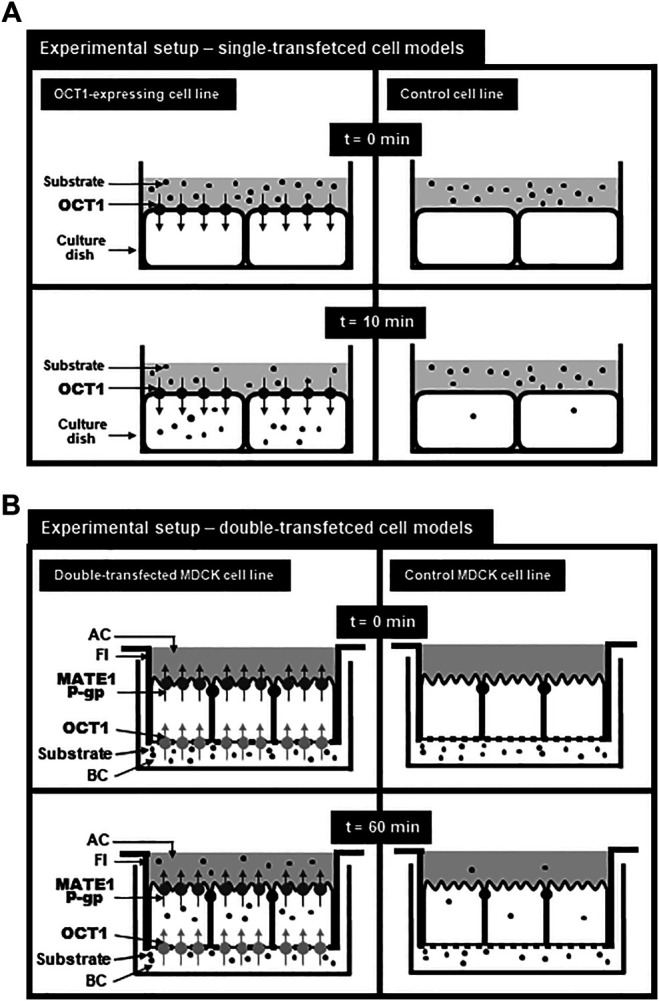
Experimental setup for using single-transfected **(A)** and double-transfected **(B)** cell models modified from Taghikhani et al. (Taghikhani et al., 2017). **(A)**: Setup for analyzing the transport function of OCT1 in single-transfected cell lines. At time point 0 min, the donor solution containing the substrate is applied onto the cell layer and after 10 min, the uptake of the substrate into OCT1-expressing cells and into control cells can be determined. By subtracting the uptake into the control cell line from the uptake into the OCT1-expressing cell line, the so called net uptake can be calculated referring to the uptake mediated by recombinantly expressed OCT1. **(B)**: Setup for vectorial transport assays using double-transfected MDCK cell lines expressing OCT1 in the basolateral membrane and MATE1 or P-glycoprotein in the apical membrane. MDCK cells were cultured on filter inserts (FI) separating a basolateral (BC) from an apical (AC) compartment. The substrate was added to the basolateral compartment and after 60 min the substrate concentration in the cells (uptake) or in the apical compartment (vectorial transport) can be calculated and compared to the uptake or the vectorial transport of the control MDCK cell line. Net intracellular substrate concentrations reflects OCT1-mediated substrate uptake and the net substrate concentration in the apical compartment reflects the vectorial transport mediated by OCT1-mediated uptake and MATE1-or P-gp-mediated export.

This article focuses on transport data of the SLC22 family member OCT1 (gene symbol *SLC22A1*) generated by different *in vitro* cell models. OCT1 is predominantly expressed in liver and localized in the basolateral membrane of human hepatocytes (Gorboulev et al., 1997; Nies et al., 2008). It mediates the uptake of several endogenous and exogenous compounds and drugs (Table 1). Single-transfected cell models (e.g., HEK-OCT1 cells) recombinantly overexpressing OCT1 were established to study OCT1-mediated transport, to calculate transport parameters (e.g., K_m_ values), to investigate the impact of genetic variations and to evaluate OCT1-mediated drug-drug interactions *in vitro* (Figure 1A; Table 1). Since OCT1 has an overlapping substrate spectrum with the apically localized export proteins MATE1 [gene symbol *SLC47A1* (Nies et al., 2011)] and P-glycoprotein [P-gp, MDR1; gene symbol *ABCB1* (Nies et al., 2008; Misaka et al., 2016)], double-transfected cell models have been established (MDCK-OCT1-MATE1 or MDCK-OCT1-P-gp) for investigating the vectorial transport mediated by both proteins (Table 2). MATE1 and P-glycoprotein are both localized in the apical (canalicular) membrane of human hepatocytes and responsible for the export of substances out of the cells into bile (Thiebaut et al., 1987; Otsuka et al., 2005). When expressed together with OCT1 in MDCK cells grown as a monolayer, OCT1 localizes in the basolateral and MATE1 or P-gp in the apical membrane (Figure 1B). In this experimental setup, substrates of OCT1 and MATE1/P-gp applied to the basolateral compartment will be first taken up into the cells mediated by OCT1 and subsequently exported via MATE1 or P-gp into the apical compartment (Figure 1B). Therefore, these cell models can be used to study not only OCT1-mediated uptake into the cells, but also the vectorial transport of substances from the basolateral into the apical compartment mimicking the transport processes during the hepatobiliary elimination e.g. of drugs (Taghikhani et al., 2017). Moreover, the importance of uptake and efflux transporters for perpetrator disposition can be assessed (Müller et al., 2018b). In this review, we summarize transport data related to the hepatocellular uptake transporter OCT1 obtained by studies in different cell models. Furthermore, the advantages and disadvantages of these cell models will be addressed.

**TABLE 1:** Substrates of OCT1 (drugs, drug metabolites, endogenous molecules, chemicals) studied in single-transfected cell lines.

Drug/Compound	Cell model	K_m_ [µM]	Concentration* [µM]	Reference
1-(2-phenoxyethyl)-biguanide	HEK293		100	Obianom et al. (2017)
1-(3-phenylpropyl)-biguanide	HEK293		100	Obianom et al. (2017)
1-(4-Phenyl-butyl)-biguanide	HEK293		100	Obianom et al. (2017)
1-(*m*-phenoxyphenyl)-biguanide	HEK293		100	Obianom et al. (2017)
1-(*p*-chlorophenethyl)-biguanide	HEK293		100	Obianom et al. (2017)
1-(*p*-chlorophenyl)-biguanide	HEK293		100	Obianom et al. (2017)
1-(*p*-methoxybenzyl)-biguanide	HEK293		100	Obianom et al. (2017)
1-(p-methyl)-biguanide	HEK293		100	Obianom et al. (2017)
1-[p-(*p*-phenoxy)phenyl]-biguanide	HEK293		100	Obianom et al. (2017)
^13^1I-labeled *m*-iodobenzylguanidine	HEK293		37 kBq	Kobayashi et al. (2020)
1-methyl-4-phenylpyridinium (MPP^+^)	*Xenopus* oocytes	14.6 ± 4.39		Zhang et al. (1997)
1-methyl-4-phenylpyridinium (MPP^+^)	HEK293	32		Gründemann et al. (2003)
1-methyl-4-phenylpyridinium (MPP^+^)	HEK293	25.0		Umehara et al. (2007)
1-n-pentylbiguanide	HEK293		100	Obianom et al. (2017)
2-(2,4-dichlorophenyl)ethyl-biguanide	HEK293		100	Obianom et al. (2017)
2-(4-biphenyl)ethyl-biguanide	HEK293		100	Obianom et al. (2017)
2,2-diphenylethyl-biguanide	HEK293	14 ± 2.8		Obianom et al. (2017)
2,3-dihydro-1H-inden-2-yl-biguanide	HEK293		100	Obianom et al. (2017)
2-ehylidene-1,5-dimethyl-3,3-diphenylpyrrolidine (EDDP)	HEK293		1	Campbell et al. (2015)
3-methoxymorphinan	HEK293		0.05–0.5	Meyer et al. (2019)
4-4-dimethylaminostyryl-N-methylpyridinium (ASP^+^)	HEK293	2.32 ± 0.29		Ahlin et al. (2008)
4-4-dimethylaminostyryl-N-methylpyridinium (ASP^+^)	HEK293	21.2		Chen et al. (2017a)
4H-1-benzopyran-4-one-biguanide	HEK293		100	Obianom et al. (2017)
Acebutol-(R)	HEK293	19.9 ± 5.7		Jensen et al. (2020b)
Acebutol-(S)	HEK293	21.0 ± 2.5		Jensen et al. (2020b)
Acetylcholine	*Xenopus* oocytes		5	Lips et al. (2005)
Aciclovir	S2	151.2 ± 22.1		Takeda et al. (2002)
Aflatoxin B1	S2		0.1	Tachampa et al. (2008)
Albuterol	HEK293		2.5	Hendrickx et al. (2013)
Amifampridine	HEK293	508.1 ± 247.3		Jensen et al. (2021)
Amiloride	HEK293		2.5	Hendrickx et al. (2013)
Amisulpride	HEK293	31.3 ± 5.4		Dos Santos Pereira et al. (2014)
Anisodine	HEK293		1–5	Chen et al. (2019)
AR-H067637	HEK293	26		Matsson et al. (2013)
AR-H069927	HEK293	116		Matsson et al. (2013)
Atenolol	MDCK	3080		Mimura et al. (2015)
Atenolol racemate	HEK293		2.5	Hendrickx et al. (2013)
Atenolol-(R)	HEK293		2.5	Hendrickx et al. (2013)
Atenolol-(R)	HEK293	201.9 ± 33.1		Jensen et al. (2020b)
Atenolol-(S)	HEK293		2.5	Hendrickx et al. (2013)
Atenolol-(S)	HEK293	196.4 ± 23.1		Jensen et al. (2020b)
Atropine	HEK293	5.9 ± 1.4		Chen et al. (2017b)
Azidoprocainamide	*Xenopus* oocytes	100.9 ± 43.0		van Montfoort et al. (2001)
Benzyltriethylammonium	HEK293	38.6 ± 9.9		Jensen et al. (2021)
Berberine	MDCK	14.8 ± 3.3		Nies et al. (2008)
Berberrubine	MDCK	1.27 ± 0.23		Li et al. (2016)
Bromosulfophthalein	HEK293	13.6 ± 2.6		Boxberger et al. (2018)
Butylscopolamine	HEK293	23.4 ± 2.3		Chen et al. (2017b)
Cimetidine	HEK293		2.5	Hendrickx et al. (2013)
*cis*-Diammine (pyr-idine)chloroplatinum(II) (cDPCP)	MDCK		10	Lovejoy et al. (2008)
Cisplatin	HEK293		1000	Yonezawa et al. (2006)
Clidinium	HEK293		2.5	Hendrickx et al. (2013)
Coptisine	MDCK	5.80 ± 1.0		Li et al. (2016)
Cyclo(His-pro)	HEK293	655 ± 191		Taubert et al. (2007)
Cycloguanil	HEK293		100	van der Velden et al. (2017)
Cycloguanil	HEK293	18.3		Matthaei et al. (2019)
DAPI	MDCK	8.94 ± 1.26		Yasujima et al. (2011)
Debrisoquine	HEK293		1	Seitz et al. (2015)
Debrisoquine	HEK293	5.9 ± 1.5		Saadatmand et al. (2012)
Debrisoquine	HEK293	24.2 ± 1.3		Neul et al. (2021)
Dehydrocordaline	MDCK	11.29 ± 3.3		Chen et al. (2020)
Denatonium	HEK293	12.6 ± 1.0		Jensen et al. (2021)
Dextrorphan	HEK293		0.05	Meyer et al. (2019)
Dimethylphenylpiperazinium	HEK293	62.0 ± 23.3		Jensen et al. (2021)
Dobutamine	HEK293	28.4 ± 16.8		Jensen et al. (2021)
Dopamine	HEK293		100	Boxberger et al. (2014)
Edrophonium	HEK293	26.4 ± 9.1		Jensen et al. (2021)
Epiberberine	MDCK	4.37 ± 0.42		Li et al. (2016)
Ethambutol	HEK293	526 ± 15.6		Parvez et al. (2018)
Ethambutol	HEK293	686		te Brake et al. (2016)
Ethidium	CHO and HEK293	0.8 ± 0.2		Lee et al. (2009)
Etilefrine-(R)	HEK293	232.9 ± 29.8		Jensen et al. (2020b)
Etilefrine-(S)	HEK293	214.0 ± 24.9		Jensen et al. (2020b)
Famotidine	HEK293	35.7 ± 7.3		Jensen et al. (2021)
Fenoterol	HEK293		2.5	Hendrickx et al. (2013)
Fenoterol	HEK293	1.78 ± 0.16		Tzvetkov et al. (2018)
Fenoterol	HEK293	2.9		Morse et al. (2020)
Fenoterol-(R,R)	HEK293	1.7 ± 0.3		Jensen et al. (2020b)
Fenoterol-(S,S)	HEK293	0.8 ± 0.2		Jensen et al. (2020b)
Fenpiverinium	HEK293	8.6 ± 3.2		Jensen et al. (2021)
Formoterol	HEK293		2.5	Hendrickx et al. (2013)
Formoterol-(R,R)	HEK293	28.3 ± 6.2		Jensen et al. (2020b)
Formoterol-(S,S)	HEK293	19.1 ± 2.0		Jensen et al. (2020b)
Frovatriptan	HEK293	61.9 ± 10.3		Jensen et al. (2021)
Furaminidine	CHO	6.1 ± 1.1		Ming et al. (2009)
Ganciclovir	S2	516.2 ± 70.3		Takeda et al. (2002)
Glycopyrrolate	HEK293		2.5	Hendrickx et al. (2013)
Guanfacine	HEK293	8.6 ± 6.1		Jensen et al. (2021)
Hydromorphone	HEK293	56.1 ± 19.1		Meyer et al. (2019)
Imeglimin	HEK293	1130		Chevalier et al. (2020)
Ipratropium	HEK293		2.5	Hendrickx et al. (2013)
Ipratropium	HEK293	13.6 ± 1.3		Chen et al. (2017b)
Jatrorrhizine	MDCK	4.46 ± 0.4		Li et al. (2016)
Jatrorrhizine	HEK293	4.94 ± 0.55		Liang et al. (2020)
Ketamine	MDCK	73.9 ± 15.2		Keiser et al. (2018)
Lamivudine	CHO	1250 ± 100		Minuesa et al. (2009)
Lamivudine	HEK293	249 ± 51		Jung et al. (2008)
Lamivudine	HEK293	786 ± 84		Arimany-Nardi et al. (2016)
Lamotrigin	KCL22		5	Dickens et al. (2012)
Mepenzolate	HEK293		2.5	Hendrickx et al. (2013)
Meptazinol	HEK293		0.1–0.5	Meyer et al. (2019)
*meta*-iodobenzylguanidine (*m*IBG)	HEK293	15.9 ± 5.3		Jensen et al. (2021)
*meta*-iodobenzylguanidine (*m*IBG)	HEK293	19.5 ± 6.9		López Quiñones et al. (2020)
Metformin	HEK293	1470 ± 190		Kimura et al. (2005)
Metformin	CHO	2160 ± 360		Nies et al. (2009)
Methylnaltrexone	HEK293	20.3 ± 5.6		Meyer et al. (2019)
Methylscopolamine	HEK293	23.4 ± 4.0		Jensen et al. (2021)
Milnacipran	HEK293	2.26 ± 1.43		Jensen et al. (2021)
Monocrotaline	HEK293		1	Seitz et al. (2015)
Monocrotaline	HEK293	109.1 ± 17.8		Chen et al. (2019)
Monocrotaline	MDCK	25.0 ± 6.7		Tu et al. (2013)
Morphine	HEK293		0.05–0.5	Meyer et al. (2019)
Morphine	HEK293		0.2	Zhu et al. (2018)
Morphine	HEK293		1	Seitz et al. (2015)
Morphine	HEK293	3.4 ± 0.3		Tzvetkov et al. (2013)
N^1^-methylnicotinamide	*Xenopus* oocytes		300	Gorboulev et al. (1997)
Nadolol	HEK293		1–1000	Misaka et al. (2016)
Naratriptan	HEK293		1000	Matthaei et al. (2016)
N-ethyllidocaine	HEK293	51.4 ± 15.4		Jensen et al. (2021)
Nitidine	MDCK	0.797 ± 0.17		Li et al. (2014)
Nizatidine	HEK293		2.5	Hendrickx et al. (2013)
N-methyladenosine	HEK293		100	Miyake et al. (2019)
N-methylquinidine	*Xenopus* oocytes	11.5 ± 2.1		van Montfoort et al. (2001)
N-methylquinine	*Xenopus* oocytes	19.5 ± 7.3		van Montfoort et al. (2001)
Norfentanyl	HEK293	7.7 ± 0.8		Meyer et al. (2019)
Norlevorphanol	HEK293		0.05–0.5	Meyer et al. (2019)
Noroxycodone	HEK293	20.05 ± 6.5		Meyer et al. (2019)
Norphenylephrine	HEK293	994.1 ± 316.5		Jensen et al. (2021)
Octopamine	HEK293	388.6 ± 246.4		Jensen et al. (2021)
O-desmethyl tramadol	HEK293		1	Tzvetkov et al. (2011)
Orciprenaline-(R)	HEK293	780.5 ± 285.9		Jensen et al. (2020b)
Orciprenaline-(S)	HEK293	808.8 ± 292.6		Jensen et al. (2020b)
Oxaliplatin	MDCK		10	Lovejoy et al. (2008)
Oxaliplatin	HEK293		1000	Yonezawa et al. (2006)
Oxibutynin	HEK293	8.82 ± 0.44		Wenge et al. (2011)
Oxophenomium	HEK293		2.5	Hendrickx et al. (2013)
Oxymorphone	HEK293		0.05	Meyer et al. (2019)
p-(3-Aminoguanidino)-benzoic acid	HEK293		100	Obianom et al. (2017)
*para*-Aminosalicylic acid	HEK293	20.3 ± 4.6		Parvez et al. (2017)
*para*-Hydroxymethamphetamine	HEK293	14.5 ± 8.7		Wagner et al. (2017)
Pazopanib	HEK293	3.47		Ellawatty et al. (2018)
Pentamidine	CHO	36.4 ± 8.3		Ming et al. (2009)
Phenformin	HEK293		100	Obianom et al. (2017)
Phenylephrine	HEK293	221.2 ± 60.3		Jensen et al. (2021)
Picoplatin	HEK293		10	More et al. (2010)
Pirbuterol-(R)	HEK293	75.3 ± 11.4		Jensen et al. (2020b)
Pirbuterol-(S)	HEK293	72.9 ± 12.3		Jensen et al. (2020b)
Prenalterol	HEK293	13.3 ± 3.4		Jensen et al. (2021)
Procainamide	HEK293		2.5	Hendrickx et al. (2013)
Procaterol	HEK293		2.5	Hendrickx et al. (2013)
Proguanil	HEK293	17.7		Matthaei et al. (2019)
Proguanil	HEK293	8.1 ± 1.6		van der Velden et al. (2017)
Prostaglandin E_2_	S2	0.66		Kimura et al. (2002)
Prostaglandin F_2α_	S2	0.48		Kimura et al. (2002)
Prothionamide	HEK293	805.8 ± 23.4		Parvez et al. (2018)
Quercetin	HEK293	2.2 ± 0.2		Glaeser et al. (2014)
Ractopamine	HEK293	2.1 ± 0.76		Jensen et al. (2021)
Ranitidine	HEK293		1	Bi et al. (2019)
Ranitidine	HEK293		2.5	Hendrickx et al. (2013)
Ranitidine	HEK293	62.9 ± 4.32		Meyer et al. (2017)
Ranitidine	*Xenopus* oocytes	70 ± 9		Bourdet et al. (2005)
Retrorsine	MDCK		1	Tu et al. (2014)
Rhodamine 123	HEK293	0.54 ± 0.21		Jouan et al. (2014)
Ritodrine	HEK293	1.67 ± 0.21		Jensen et al. (2021)
Rizatriptan	HEK293		1000	Matthaei et al. (2016)
Salbutamol	HEK293		0.03–10	Salomon et al. (2015)
Salbutamol-(R)	HEK293	224.2 ± 18.4		Jensen et al. (2020b)
Salbutamol-(S)	HEK293	222.5 ± 20.5		Jensen et al. (2020b)
Salsolinol	HEK293	440 ± 209		Taubert et al. (2007)
Saracatinib	HEK293		10	Harrach et al. (2017)
Sematilide	HEK293	102 ± 24.6		Jensen et al. (2021)
Serotonin	HEK293	197 ± 42		Boxberger et al. (2014)
Sorafenib	CHO	3,8		Swift et al. (2013)
Sotalol	HEK293	195.9 ± 72.1		Jensen et al. (2021)
Sparteine	HEK293	27.2 ± 2.8		Neul et al. (2021)
Sulpiride	HEK293	259.7 ± 5.4		Dos Santos Pereira et al. (2014)
Sulpiride	HEK293	2.57 ± 0.64		Takano et al. (2017)
Sumatriptan	HEK293		2.5	Hendrickx et al. (2013)
Sumatriptan	HEK293	46		Morse et al. (2020)
Sumatriptan	HEK293	55.4 ± 7.8		Matthaei et al. (2016)
Terbutaline	HEK293		2.5	Hendrickx et al. (2013)
Tetraethylammonium (TEA)	*Xenopus* oocytes		100	Zhang et al. (1997)
Tetraethylammonium (TEA)	HEK293	140		Hendrickx et al. (2013)
Tetraethylammonium (TEA)	HeLa	164 ± 17.9		Bednarczyk et al. (2003)
Tetraethylammonium (TEA)	MDCK	1750 ± 70		Yasujima et al. (2011)
Tetraethylammonium (TEA)	HeLa	229 ± 78.4		Zhang et al. (1998)
Tetraethylammonium (TEA)	HEK293	69.2		Umehara et al. (2007)
Thiamine	HEK293		1	Bi et al. (2019)
Thiamine	HEK293		0.025	Liang et al. (2018)
Thiamine	HEK293	780 ± 64		Chen et al. (2014)
Thiamine	HEK293	1997 ± 174		Jensen et al. (2020a)
Tiotropium	HEK293		2.5	Hendrickx et al. (2013)
Tributylmethylammonium	*Xenopus* oocytes	53.0 ± 13.9		van Montfoort et al. (2001)
Trimethylamine N-oxide	HEK293	33900 ± 2700		Miyake et al. (2017)
Tropisetron	HEK293		1	Tzvetkov et al. (2012)
Tropisetron	HEK293		1	Seitz et al. (2015)
Trospium	HEK293	106 ± 16		Bexten et al. (2015)
Trospium	HEK293	15.1 ± 3.1		Chen et al. (2017b)
Trospium	MDCK	22.0 ± 3.0		Deutsch et al. (2019)
Trospium	HEK293	17.0 ± 4.64		Wenge et al. (2011)
Tyramine	HEK293	94.7 ± 28.2		Seitz et al. (2015)
Xamoterol (R)	HEK293		2.5	Hendrickx et al. (2013)
Xamoterol (S)	HEK293		2.5	Hendrickx et al. (2013)
YM155	HEK293	22.1 ± 2.5		Minematsu et al. (2010)
YM155	S2	38.7		Iwai et al. (2009)
Zalcitabine	HEK293	242 ± 56		Jung et al. (2008)
Zolmitriptan	HEK293		1000	Matthaei et al. (2016)

Concentration* = substance was tested using the stated concentration with an uptake rate ≥2‐fold compared to the uptake into control cells.

**TABLE 2:** OCT1 expressing, double-transfectant cell lines and investigated substrates and inhibitors.

Cell system	Expressed proteins	Working group establishing these cells	References	Tested substrates	Inhibitors
MDCK	OCT1 and P-gp	Nies et al.	Nies et al. (2008)	Berberine, TEA, MPP^+^	LY335979
		König et al.	Misaka et al. (2016)	Berberine, nadolol	Zosuquidar
	OCT1 and MATE1	Sato et al.	Sato et al. (2008)	TEA, MPP^+^, metformin, cimetidine, creatinine, guanidine, procainamide, quinidine	MPP^+^, levofloxacine
		König et al.	König et al. (2011)	MPP^+^, metformin	
			Reznicek et al. (2017)	Emtricitabine	Cimetidin, ritonavir
			Chen et al. (2017b)	Ipratropium	
			Deutsch et al. (2019)	Trospium	
			Ceckova et al. (2016)	Lamivudine	Mitoxantrone
			Ceckova et al. (2018)	MPP^+^, lamivudine	Efavirenz
		Li et al.	Li et al. (2016); Li et al. (2018)	Metformin	Nuciferine
	OCT1 and CYP3A4	Tu et al.	Tu et al. (2014)	ASP^+^, Retrorsine	TEA
HEK293	OCT1 and MATE1	van der Velden et al.	van der Velden et al. (2017)	Proguanil	
LLC-PK1	OCT1 and P-gp	Iwai et al.	Iwai et al. (2011)	YM155, digoxin	YM155, digoxin, cyclosporin A

## Organic Cation Transporter 1 and Related Export Proteins

### Organic Cation Transporter 1

The rodent orthologue of human OCT1 (rOct1) was first isolated from a rat kidney library and expressed in *Xenopus* oocytes. This rOct1 transporter showed inhibitable and potential-dependent Tetraethylammonium (TEA) and 1-methyl-4-phenylpyridinium (MPP^+^) uptake (Gründemann et al., 1994). Additionally, *in situ* hybridization and northern blotting analysis demonstrated Oct1 expression in rat hepatocytes and enterocytes. In 1997, human OCT1 (gene symbol: *SLC22A1*) was cloned and characterized by two independent working groups (Gorboulev et al., 1997; Zhang et al., 1997). Although Gorbulev et al. amplified hOCT1 using kidney cDNA, northern blot analysis demonstrated OCT1 expression mainly in the liver (Gorboulev et al., 1997), which was in line with the findings of Zhang et al. using liver cDNA (Zhang et al., 1997). Later, OCT1 was localized at the basolateral membrane of human hepatocytes (Nies et al., 2008). OCT1 facilitates the uptake of organic cations or weak bases (Table 1), which comprises approximately 40–67.5% of all drugs (Comer and Tam, 2001; Neuhoff et al., 2003; Manallack, 2007), into human hepatocytes. In the 2018 recommendations of the International Transporter Consortium (ITC), the investigation of OCT1-mediated transport during drug development was added, based on clinically important OCT1-mediated drug-drug interactions (Zamek-Gliszczynski et al., 2018a; Zamek-Gliszczynski et al., 2018b).

### Multidrug and Toxin Extrusion Protein 1

The existence of an organic cation-H^+^ antiporter was already postulated back in 1985 by studying the transport of N^1^-methylnicotinamide by the use of membrane vesicles, derived from the brush border membrane of rabbit kidney (Wright, 1985; Inui et al., 2000). The multidrug and toxic compound extrusion family (MATE) was first characterized in bacteria (Pallen, 1999) and Otsuka et al. (Otsuka et al., 2005) identified human and mouse orthologues of the bacterial MATE protein by genomic databank screening. The human MATE family consists of two members, the more widely expressed MATE1 protein and the kidney-specific member MATE2-K. The MATE1 protein is localized in the apical membrane of kidney proximal tubule epithelial cells and in the canalicular membrane of human hepatocytes (Otsuka et al., 2005; Masuda et al., 2006). MATE1 substrates are cations or have a positively charge at physiological pH (Nies et al., 2016). MATE proteins have a strong substrate overlap with the SLC22 family members OCT1, OCT2 and OCT3, indicating an interplay between these transporters in the hepatobiliary and renal elimination of drugs and endogenous compounds. The ITC recommends *in vitro* uptake studies using MATE-transfected cells, if the new molecular entity (NME) shows renal secretion as route of elimination or if the NME is an inhibitor of MATE1/2-K or OCT2 (Hillgren et al., 2013). So far, no criteria are defined for the evaluation of hepatic elimination of drugs mediated by MATE1. Detailed lists of substrates and inhibitors are available in several reviews (Terada and Inui, 2008; Damme et al., 2011; Nies et al., 2011; Motohashi and Inui, 2013; Nies et al., 2016; Koepsell, 2020).

### P-glycoprotein

P-glycoprotein (P-gp) is an ABC transporter and acts as an efflux pump for a variety of drugs such as digoxin, dabigatran etexilate and indinavir. P-gp is due to its ability of extruding drugs an limiting factor for drug bioavailability (Fromm, 2004). The substrate spectrum shows a strong overlap with the substrates of the Cytochrome P450 enzyme CYP3A4 and both proteins together protect the organism from xenobiotics (Kivistö et al., 2004; von Richter et al., 2004). P-gp is expressed in the apical membrane of several tissues such as small intestine, liver and kidney (Thiebaut et al., 1987). Additionally, P-gp plays an important role at blood-tissue barriers such as the blood-brain barrier and placenta, protecting the central nervous system or the unborn child from drugs or other xenobiotics (Fromm, 2004). Furthermore, P-gp is overexpressed in several cancer tissues, leading to multidrug resistance (Gottesman et al., 2002; Leopoldo et al., 2019). Wang et al. (Wang et al., 2003) analyzed by structure activity relationship analysis (SAR) several substrates and inhibitors of P-gp. They postulated that a tertiary nitrogen atom could be beneficial for the binding to P-gp due to the stronger interaction of the formed cation with the binding sites of P-gp. These cationic properties of some P-gp substrates already indicate that there might be an interplay between the OCT1-mediated uptake and the P-gp-mediated efflux during hepatobiliary elimination. Based on the recommendations of the ITC and FDA (International Transporter Consortium et al., 2010; Food and Drug Administration, 2020), a NME should be tested as P-gp substrate using inside-out oriented membrane vesicles or by vectorial transport assays using polarized grown cell lines such as Caco-2 cells or cell lines (MDCK, LLC-PK_1_) recombinantly overexpressing P-gp.

## Cell Models to Study Organic Cation Transporter 1 Transport Function

### Single-Transfected Cell Models for Investigating Organic Cation Transporter 1

Use of single-transfected cell models expressing the transporter of interest is often the first step to gain insights into the substrate spectrum. The transporter is either transiently or stably transfected into a suitable cell line. The most commonly used cell lines for uptake studies are Human Embryonic Kidney 293 cells (HEK293). HEK293 cells are easy to culture and have, due to their human origin, comparable posttranslational protein modification to human tissues (Hu et al., 2018). Additionally, after transfection HEK293 cells are capable of expressing a variety of different proteins (Thomas and Smart, 2005). To study transport proteins, uptake assays can be used to determine transport parameters (K_m_ or C_max_ values) of the selected substrate (Figure 1A) or to perform drug-interaction studies. One limitation of using HEK293 cells is the lack of polarized growth, which excludes them for the analysis of transcellular transport studies. Other frequently used cell lines for establishing single-transfected cell models with the expression of one transport protein are Madin-Darby Canine Kidney cells (MDCK), Chinese Hamster Ovary cells (CHO), *Drosophila* Schneider 2 cells (S2), HeLa cells and *Xenopus* oocytes. *Xenopus* oocytes are a robust cell model, which is derived from *Xenopus laevis* (Zeng et al., 2020). The exogenous mRNA encoding the transport protein of interest is injected into oocytes leading to a functional expression of the protein. However, because of their limited longevity *Xenopus* oocytes cannot be used to generate stable transfectants.

Pioneering work on the characterization of OCT1 was done by Zhang et al. (1997). They were the first to clone OCT1 from human liver and they used *Xenopus* oocytes to analyze OCT1-mediated transport. They calculated the first transport K_m_ and V_max_ parameters for the uptake of the organic cation MPP^+^ and measured the IC_50_ values for the inhibition of OCT1-mediated transport of MPP^+^ by the cations decynium-22, vecuronium and TEA (Zhang et al., 1997). Furthermore, they extended their research by using transiently transfected HeLa cells and characterized the transport of TEA and obtained IC_50_ values for 15 different compounds (Zhang et al., 1998). The first inhibitor analysis using a wide range of compounds was done by Bednarczyk et al. (Bednarczyk et al., 2003). They used OCT1-transfected HeLa cells and calculated IC_50_ values of 30 structurally diverse organic cations and established a model of inhibitor/OCT1 interaction (Bednarczyk et al., 2003). These findings of structural requirements for OCT1 inhibition were extended by Ahlin and coworkers and their analysis of the inhibitory effect of 191 compounds on the OCT1-mediated uptake of ASP^+^ (Ahlin et al., 2008). ASP^+^ [4-(4-(dimethylamino)styryl)-N-methylpyridinium] is a fluorescent cationic model substrate for OCT1, which enables the fast screening of drugs as inhibitors of OCT1-mediated transport by analyzing fluorescence uptake. They identified 62 of the investigated compounds as inhibitors (cutoff value ≥50% inhibition) of which 66% were cations, 32% were neutral and repaglinide was the only anionic compound. Therefore, they estimated that high lipophilicity and a cationic character are the two main physicochemical properties of potent OCT1 inhibitors (Ahlin et al., 2008). A detailed analysis of the ‘structure-transport relationship’ was missing until Hendrickx et al. analyzed the uptake of 354 (with 83 marketed drugs) compounds into stably transfected HEK293 cells expressing OCT1 using a LC-MS/MS approach (Hendrickx et al., 2013). TEA and ipratropium served as reference compounds. In this study, the molecular volume of a compound was identified as the best descriptor for OCT1 substrates and lipophilicity was identified to be not important (Hendrickx et al., 2013). Recent publications emphasized the use of *in silico* predictions and machine learning approaches for the identification of new OCT1 substrates and their molecular characteristics (Baidya et al., 2020; Jensen et al., 2021). The OCT1 substrate and/or inhibitor spectrum has intensively been studied by various groups [e.g., (Gorboulev et al., 1997; Ciarimboli et al., 2005; Wenge et al., 2011; Tzvetkov et al., 2013; Knop et al., 2015; Otter et al., 2017; Meyer et al., 2019; Jensen et al., 2020b; Koepsell, 2020)].

Single-transfected cell models have also been extensively used to study the influence of genetic polymorphisms in the *SLC22A1* gene on kinetic parameters of the OCT1-mediated transport (Kerb et al., 2002; Shu et al., 2003; Tzvetkov et al., 2011; Tzvetkov et al., 2013; Dos Santos Pereira et al., 2014; Matthaei et al., 2016; Meyer et al., 2017; Jensen et al., 2020b). A detailed list about the *in vitro* analyzed effects of genetic polymorphisms in the *SLC22A1* gene has been published by Koepsell (2020). Furthermore, comparisons of human OCT1 with the orthologues of rat or mouse Oct1 has been performed using single-transfected cell models to gain insights into our understanding of potential substrate binding sites or protein regions involved in substrate recognition (Egenberger et al., 2012; Floerl et al., 2020; Koepsell, 2020; Meyer et al., 2020).

Table 1 summarizes currently known OCT1 substrates. We included all data where a K_m_-value was determined or where the uptake was ≥2-fold higher in the OCT1-expressing cells compared to the uptake into the respective control cell line. Potential substrates with uptake ratios between 1.5 and 2 are shown in Supplementary Table S1, together with publications that were not able to reproduce uptake experiments with controversial substrates (e.g., imatinib). OCT1 inhibitors are shown in Supplementary Table S2. We also included inhibition experiments, where no IC_50_ values were calculated, if the inhibitor was able to reduce the uptake of the substrate to ≤50%. Nevertheless, these lists are not exhaustive.

### Double-Transfected Cell Lines

In contrast to HEK293 cells, MDCK cells form confluent monolayers when seeded on permeable membranes, such as microplate thinserts, separating a basolateral from an apical compartment (Figure 1B). These cells can be transfected with two cDNAs, for example one cDNA encoding for a basolaterally localized uptake transporter and one cDNA for an apically localized export protein. This allows a more versatile experimental setup, because these culture conditions enable transcellular transport measurements in combination with the measurement of the intracellular accumulation of the substrates. Furthermore, substrates can be applied either to the basolateral or apical compartment mimicking both routes of substrate transport, the route of excretion with the uptake of substrates from blood across the basolateral membrane and the export across the apical membrane into bile or urine (basal to apical transport) or the route of reuptake of substances across the apical membrane and the export into the blood (apical to basolateral transport e.g., during renal reabsorption). Limitations of this cell line are the expression of endogenous canine transporters such as canine Mdr1, Mrp2 and Oct2, which may affect the transport studies. Additionally, it is absolutely necessary to investigate the tightness of the cell monolayer to avoid paracellular transport of substances (Volpe, 2011).

The first double-transfected MDCK cell line expressing human OCT1 as uptake transporter together with P-gp in the apical membrane was established by Nies et al. [MDCK-OCT1-P-gp, Table 2 (Nies et al., 2008)]. The protein expression was investigated by immunoblot and immunofluorescence analysis and for the functional testing, TEA and MPP^+^ served as prototypic substrates for OCT1. Subsequent to the identification of berberine, a quaternary isoquinoline alkaloid, as an OCT1 and OCT2 substrate, the authors used the MDCK-OCT1-P-gp cell line to analyze the transcellular transport of this substance. The transport of berberine from the basal to the apical compartment was 3-fold, 5-fold and 1-fold higher in MDCK-OCT1-P-gp cells compared to the vectorial transport measured with MDCK-OCT1 and MDCK-P-gp single-transfected cells and MCDK control cells, respectively. Furthermore, the addition of the P-gp inhibitor LY335979 resulted in a decrease of the transcellular transport to the level measured in MCDK control cells. Even though the transcellular transport could be inhibited, an increase of the intracellular berberine amount was observed in MDCK-OCT-P-gp cells, indicating that LY335979 specifically inhibits the P-gp mediated export. Misaka et al. also established a MDCK-OCT1-P-gp double-transfectant and this cell line also showed a significant basal to apical transcellular transport of berberine, which could not be measured in the respective single-transfectants (Misaka et al., 2016). They also investigated the transcellular transport of nadolol (10 µM) with and without the addition of 1 µM zosuquidar, a known P-gp inhibitor, demonstrating that zosuquidar was able to significantly inhibit the basal to apical transport of nadolol (Misaka et al., 2016).

Sato et al. (Sato et al., 2008) established an OCT1-MATE1 double-transfected MDCK cell line and investigated the expression and localization by immunofluorescence microscopy. They used TEA as prototypic substrate and measured the transcellular transport from the basolateral to apical (b→a) and from the apical to basolateral (a→b) compartment demonstrating that the cellular accumulation was 66-fold higher, when TEA was applied to the basolateral compartment. Additionally, they were able to reproduce the pH-dependency of MATE1-mediated transport by varying the apical pH and demonstrated that the transcellular transport showed maximal transport rates at extracellular pH 6.5. The addition of 10 mM MPP^+^ or 1 mM levofloxacin significantly decreased the basolateral to apical transport of TEA. To further analyze the transport of organic cations, Sato and coworkers measured the transcellular transport and cellular accumulation of MPP^+^, metfomin, cimetidine, creatinine, guanidine, procainamide and quinidine and found significant vectorial transport rates for all substances, applied to the basolateral compartment. Unfortunately, they did not show a comparison between transcellular transport rates and the cellular uptake of substances into the MDCK-OCT1-MATE1 double-transfectant and into the corresponding single-transfectants (MDCK-OCT1 or MDCK-MATE1). The importance of the interplay of OCT1 and MATE1, studied in double-transfected cell lines could also be demonstrated by Sato et al. (Sato et al., 2008). Experiments using HEK293 cells transfected with OCT1 only showed slightly higher uptake rates of quinidine and procainamide (<2 fold) and the HEK-MATE1 cell line showed small uptake rates for quinidine (<2 fold) compared to the uptake into the vector control cell lines. This is contradictory to *in vivo* data that had already shown that quinidine (Notterman et al., 1986) and procainamide (Somogyi et al., 1983) are secreted renally. This underestimation of the role of OCT1 and MATE1 for the transport of both substrates was abolished by the use of double-transfected cell lines where significant transcellular transport rates could be measured for procainamide as well as for quinidine (Sato et al., 2008).

Our working group extended the investigations of Sato et al. by also establishing a MDCK-OCT1-MATE1 double-transfectant (König et al., 2011). The corresponding single-transfected cell lines (MDCK-OCT1 and MDCK-MATE1) were also used for transport assays. The cellular accumulation of MPP^+^ (10 and 50 µM) and metformin (10 and 50 µM) was highest in MDCK-OCT1 single-transfected cells. Interestingly, the lowest intracellular accumulation was measured in the MDCK-MATE1 single-transfected cells and not in the MDCK control cells. This can be explained by MATE1-mediated efflux of MPP^+^ or metformin taken up by an endogenous transporter or diffused passively into the cells when applied to the basolateral compartment. Intracellular accumulation in the MDCK-OCT1-MATE1 double-tranfected cell line was also significantly higher compared to the accumulation in the MDCK control cell line demonstrating OCT1-mediated uptake. As expected, there was no significant difference in the transcellular transport of the single-transfected cell lines and the MDCK control cells. In contrast, the MDCK-OCT1-MATE1 double-transfectant showed significantly higher transcellular transport rates for both substrates (10-fold basal to apical over apical to basal transcellular transport of metformin after 60 min). In the following years, several publications used double-transfected OCT1-MATE1 cell models to gain more insights into vectorial transport of organic cations. Reznicek et al. (Reznicek et al., 2017) used emtricitabine as substrate for vectorial transport studies and demonstrated that the transcellular transport is independent of OCT1-mediated uptake. This transport was saturable at very high concentrations (1 mM), temperature- and pH-dependent (decreasing the apical pH significantly increased the b→a transcellular transport). Furthermore, the addition of cimetidine and ritonavir, both known MATE1 inhibitors, resulted in an inhibition of the transcellular transport of emtricitabine by 43 and 35% in the double-transfectant, whereas the intracellular accumulation increased to 143 and 135%, respectively.

Chen et al. (Chen et al., 2017b) demonstrated that the basal to apical transcellular transport of ipratropium (0.5 µM) was 9.9-fold higher in MDCK-OCT1-MATE1 double-transfected cells compared to control cells and Deutsch and colleagues (Deutsch et al., 2019) identified trospium as substrate for both transporters using the same transporter combination. The vectorial basal to apical transport of trospium (1 µM) was 24.5-fold higher compared to the vectorial transport in the control cell line. As expected, the transcellular transport was highest at extracellular pH 6.5, whereas intracellular accumulation was lowest at this pH, demonstrating that OCT1 and MATE1 play an important role in the transcellular transport of trospium.

Ceckova et al. (Ceckova et al., 2016) analyzed the transcellular transport and intracellular accumulation of lamivudine in MDCK-OCT1-MATE1 double-transfected cells and their respective control and single-transfected cell lines. The transcellular transport (b→a) measured in the MDCK-MATE1 and MDCK-OCT1-MATE1 cells was significantly higher in comparison to the MDCK control cells and to the MDCK-OCT1 single-transfectant, whereas the intracellular accumulation of lamivudine was the highest in the MDCK-OCT1 cell line. This transcellular transport could be inhibited by the simultaneous application of lamivudine and mitoxantrone (2 µM) to the basolateral compartment and was reduced to a level which was not significantly different to the MDCK control cells. The fact, that mitoxantrone inhibition led to an increase of the intracellular accumulation of lamivudine, underlines the importance of MATE1 on the transport of lamivudine. Later, Ceckova et al. (Ceckova et al., 2018) used the MDCK-OCT1-MATE1 double-transfectant to study the inhibition of the transcellular transport of 2 nM MPP^+^ and 10 nM lamivudine by adding efavirenz. In both cases, the presence of 10 µM efavirenz in the basolateral compartment reduces the basolateral to apical transport in all single- and double-transfected cell lines, except in the MDCK control cells. The intracellular accumulation of both substrates was decreased in the MDCK-OCT1 cells but increased in the MDCK-MATE1 cells, confirming the potential of efavirenz as an *in vitro* inhibitor of both transport proteins (Ceckova et al., 2018). Li et al. (Li et al., 2018) addressed a potential drug-drug interaction between metformin and nuciferine, the active ingredient of lotus leafs (*Folium Nelumbinis*). This herbal drug is used as tea or food supplement for the elderly population suffering from hyperlipidemia and therefore a concomitant use of these herbs with antidiabetic drugs seems quite likely. After the evaluation of nuciferine inhibition (0.01–100 µM) on the OCT1-and MATE1-mediated uptake of metformin (10 µM) in single-transfected cells, they verified these findings by measuring the intracellular accumulation and transcellular transport of 10 µM metformin alone and in the presence of nuciferine (5–80 µM) in the double-transfected cell line. At all investigated time points the basolateral to apical transport of metformin was significantly higher in the MDCK-OCT1-MATE1 double-transfectant, compared to the transport in the MDCK-OCT1 single-transfectant. This transport could be inhibited by adding nuciferine in a concentration-dependent manner. Furthermore, nuciferine also reduced the intracellular accumulation of metformin. In contrast, transcellular transport from the apical to the basolateral compartment was unaltered by the addition of nuciferine. This demonstrates that nuciferine is an inhibitor of both OCT1 and MATE1. Remarkably, when applying the same experimental setup to the MDCK-OCT2-MATE1 double-tranfectant, the transcellular transport of metformin was also decreased but the intracellular accumulation of metformin significantly increased in a concentration-dependent manner after addition of nuciferine. This indicates, that the inhibition of MATE1 is responsible for this effect and nuciferine inhibits OCT1, but not OCT2 (Li et al., 2018).

In an interesting experimental setup van der Velden et al. (van der Velden et al., 2017) were not using MDCK cells to establish double-tranfectants. Instead, they used single-transfected HEK293 cells expressing OCT1 and cotransfected them with MATE1 or with MATE2-K and analyzed proguanil uptake. Because of the lack of polarized growth, vectorial transport studies cannot be performed with the double-transfected HEK293 cells. There was no significant difference in the uptake rate of HEK-OCT1 cells compared to HEK-OCT1-MATE1 cells, but the HEK-OCT1-MATE2-K cells showed a significant lower intracellular accumulation of proguanil, indicating an interplay between OCT1-mediated uptake and MATE2-K-mediated export (van der Velden et al., 2017).

Double-transfected cell models cannot only be used to study the interplay of uptake and efflux transporters, but also to investigate the interplay between transport proteins and metabolizing enzymes. To investigate this, Tu et al. established a double-transfected MDCK cell line, expressing OCT1 together with the phase I drug metabolizing enzyme CYP3A4 (Tu et al., 2014). This CYP enzyme is responsible for the metabolism of approx. 50% of all marketed drugs (Zhou, 2008). They validated the mRNA expression by RT-qPCR and confirmed the OCT1-mediated uptake by using the prototypical substrate ASP^+^ with or without the presence of TEA as transport inhibitor. The MDCK-OCT1 single-transfectant as well as the MDCK-OCT1-CYP3A4 double-transfectant showed significantly higher ASP^+^ uptake rates compared to the control cell line, which was strongly reduced by the addition of TEA. The CYP3A4 function in the MDCK-OCT1-CYP3A4 cells was confirmed by a CYP3A4 metabolism activity assay and was comparable to the values determined in MDCK-CYP3A4 single-transfected cells. Subsequently, they tested the cytotoxic activity of retrorsine, a hepatotoxic pyrrolizidine alkaloid, using all established MDCK cell lines. Prior experiments showed that the uptake of retrorsine is significantly higher in MDCK-OCT1 cells compared to the uptake into the MDCK control cells. Furthermore, Fu et al. demonstrated that pyrrolizidine alkaloids exhibit cytotoxicity only after bioactivation, which is mainly mediated by CYP3A4 (Fu et al., 2004). In line with these findings, the cytotoxicity of retrorsine was highest in the MDCK-OCT1-CYP3A4 cell line because of both uptake and bioactivation. There was no difference in the cytotoxicity between control cells and MDCK-OCT1 cells, due to the missing CYP-mediated activation. The MDCK-CYP3A4 single-transfectant also exhibit significantly higher retrorsine sensitivity, but still significantly lower compared to the double-transfectant (Tu et al., 2014).

Instead of MDCK cells, Iwai et al. used Lilly Laboratory Cancer Porcine Kidney 1 cells (LLC-PK1) to establish an OCT1-P-gp double-transfected cell line (Iwai et al., 2011). LLC-PK1 cells form tight monolayers and LLC-P-gp cells are recommended by the FDA as bidirectional transcellular transport system for identifying P-gp substrates and inhibitors Food and Drug Administration (2020). OCT1 function in these double-transfected cells was confirmed by using MPP^+^ as prototypical substrate and the transport function of P-gp was verified by using digoxin as substrate. The basal to apical transcellular transport of 1-(2-methoxyethyl)-2-methyl-4,9-dioxo-3-(pyrazin-2-ylmethyl)-4,9-dihydro-1*H*-naphtho [2,3-d]imidazolium bromide (YM155, 1 µM), a survivin suppressant and known substrate of OCT1 (Iwai et al., 2009), was much higher in the LLC-OCT1-P-gp double-transfectant compared to LLC-control, LLC-OCT1 and LLC-P-gp single-tranfected cell lines, demonstrated by the high basal to apical flux ratio of 16.6. This transcellular transport decreased by adding cyclosporine A or 1 mM MPP^+^, respectively, indicating that YM155 is a substrate of both OCT1 and P-gp. The relatively high basal to apical transcellular transport of 1 µM digoxin was unaffected by the addition of 100 µM YM155 but was reduced to the level of the apical to basal transport by adding 10 µM cyclosporine A, demonstrating that YM155 has a low inhibitory effect on P-gp-mediated transport even at higher concentrations. Table 2 gives an overview about the studies using OCT1 expressing double-transfected cell lines.

## Discussion

*In vitro* cell models expressing transport proteins are useful tools for studies of transporter function and for the identification of transporter substrates and/or inhibitors. Therefore, the FDA and EMA recommend the usage of such cell lines during preclinical drug development. The FDA considers an investigational drug as an *in vitro* substrate for hepatic or renal transporters, ‘if uptake is ≥ 2-fold of the drug uptake in empty vector-transfected cells and if a known inhibitor can decrease the drug uptake to ≤50% at a concentration at least 10 times that of the K_i_ or IC_50_’. To test whether a drug is an inhibitor it is recommended to ‘determine the inhibition potency (K_i_ or IC_50_) of the drug on the uptake of a known substrate’ Food and Drug Administration (2020). In this review we describe cell models for the investigation of the SLC22 family member OCT1. Using single-transfected cell lines expressing OCT1, several drugs could be identified as substrates and inhibitors of this transporter (Table 1; Supplementary Table S2). Interestingly, it has been demonstrated that OCT1 transport inhibition is substrate-dependent. For example, Boxberger et al. detected substrate-dependent inhibition for several drug (e.g., ranitidine and fluoxetine) by using MPP^+^, serotonin and TEA as probe substrates in competitive counterflow experiments (Boxberger et al., 2018). Therefore, the use of multiple probe substrates for *in vitro* testings of OCT1 seems reasonable and the use of substrates for the inhibition analysis *in vitro* that can also be used in the subsequent clinical studies as recommended by the FDA Food and Drug Administration (2020).

Despite the frequent use of single- and double-transfected cell lines, *in vitro*-*in vivo* extrapolations (IVIVE) have still limitations. Many drugs listed in Supplementary Table S2 only inhibit the transport of substrates at concentrations above their therapeutic plasma concentration or environmentally exposed concentration so that the inhibitory potential is more theoretically relevant (Chedik et al., 2019). *In vitro* studies that analyzed opioids as inhibitors of OCT1, Meyer et al. showed that the calculated maximal unbound plasma concentrations for most of the tested opioids are lower than the obtained IC_50_ values for OCT1 mediated transport (Meyer et al., 2019). Only the maximal portal vein concentration of tapentadol was comparable to the obtained IC_50_ value, indicating a potential drug-drug interaction *in vivo* (Meyer et al., 2019). Furthermore, the influence of endogenous expression of transport proteins in the different cell lines, the use of different cell models (e.g., Table 1: K_m_ TEA determined in MDCK cells, HEK293 cells and HeLa cells) and the independent establishment of several stable transfectants by different working groups lead to interlaboratory variability in the gained K_m_ and IC_50_ values and to a limited IVIVE. The use of primary human hepatocytes after the *in vitro* validation of drugs as substrates or inhibitors of OCT1, as recommended by Bi et al., could be helpful to gain better predictions of the hepatic clearance or to identify potential DDIs and could help to evaluate the contribution of the OCT1-mediated transport of potential substrates by using selective inhibitors (Bi et al., 2019; Jensen et al., 2020a). Interestingly, strong variations in the uptake of OCT1 substrates (MPP^+^ and ASP^+^) were detected comparing human hepatocytes from different donors (De Bruyn et al., 2011; Fattah et al., 2017) and the genetic characterization revealed strong genetic variabilities between the tested batches, where 13 of 27 tested hepatocyte batches showed at least 1 nonfunctional allele of the *SLC22A1* gene (Fattah et al., 2017).

The identification of OCT1 as rate-limiting transporter in the hepatic uptake of clinical important drugs together with *in vivo* data on reported genetic effects led to the update of the ITC recommendations, where OCT1 is now mentioned as transporter of emerging clinical importance (Zamek-Gliszczynski et al., 2018b).

Double-transfected cell lines could lead to an even better understanding of vectorial transport processes during hepatobiliary and renal elimination. They allow the simultaneous measurement of more parameters and are helpful to identify the individual transport protein underlying clinically observed drug-drug interactions and to study the impact of the respective transporters on perpetrator disposition (Müller et al., 2018b). Important double-transfected cell models for investigating the role of OCT1 in the hepatobiliary elimination of drugs are MDCK-OCT1-MATE1 cells expressing OCT1 together with the apically localized export protein MATE1. Both proteins share an overlapping substrate spectrum (Nies et al., 2011) and the vectorial transport of drugs mediated by both transporters has been described (Table 2). Interestingly, only by using double-transfected cell models the direction of the MATE1-mediated transport in the double-transfected cell lines resembles the physiological direction (efflux of substrates into the apical compartment), whereas the use of MATE1-transfected HEK293 cells only allows uptake measurements into the cell. In the recent years, several working groups established double-transfected cell lines to analyze the molecular mechanisms underlying polarized transport of endogenous compounds and drugs. Moreover, they are very useful tools for the understanding of the molecular mechanisms underlying clinically relevant drug-drug interactions (Table 2).
