# Supplementary material for: Transport of Drugs and Endogenous Compounds Mediated by Human OCT1: Studies in Single- and Double-Transfected Cell Models
Source: Front Pharmacol. 2021 Apr 22;12:662535. doi: 10.3389/fphar.2021.662535 (PMC8100673; doi:10.3389/fphar.2021.662535)
Supplement: Supplementary file 1 [file table1.docx]

Tab S1. Potential substrates of OCT1 and substrates with conflicting data

| **Drug/Compound** | **Cell model** | **Tested conc. [µM]** | **Notes** | **Reference** |
| --- | --- | --- | --- | --- |
| 3-heptylbenzyl-biguanide | HEK293 | 100 | Uptake ratio between 1.5 and 2 | (Obianom et al., 2017) |
| Agmatine | HEK293 | 0.1 | Uptake ratio between 1.5 and 2 | (Gründemann et al., 2003) |
| Aminoguanidine | HEK293 |  | No comparison vs. Mock | (Kimura et al., 2009) |
| Atropine | HEK293 | 2.5 | Uptake ratio between 1.5 and 2 | (Hendrickx et al., 2013) |
| Benzamil | HEK293 | 2.5 | Uptake ratio between 1.5 and 2 | (Hendrickx et al., 2013) |
| Camostat | HEK293 | 2.5 | Uptake ratio between 1.5 and 2 | (Hendrickx et al., 2013) |
| Creatinine | HEK293 |  | not a substrate | (Urakami et al., 2004) |
| Creatinine | S2 |  | not a substrate | (Imamura et al., 2011) |
| Creatinine | HEK293 |  | not a substrate | (Ciarimboli et al., 2012) |
| Creatinine | MDCK |  | not a substrate | (Lepist et al., 2014) |
| Daunorubicin | HEK293 | 5 | Uptake ratio between 1.5 and 2 | (Andreev et al., 2016) |
| Disopyramide | HEK293 | 2.5 | Uptake ratio between 1.5 and 2 | (Hendrickx et al., 2013) |
| Hydrocodone | HEK293 | 0.1 | Uptake ratio between 1.5 and 2 | (Meyer et al., 2019) |
| Imatinib | KCL22 | 5 | Uptake ratio between 1.5 and 2 | (Wang et al., 2008) |
| Imatinib | *Xenopus* oocytes |  | not a Substrate | (Blanc Mettral et al., 2019) |
| Imatinib | HEK293 |  | not a Substrate | (Blanc Mettral et al., 2019) |
| Imatinib | *Xenopus* oocytes |  | not a Substrate | (Nies et al., 2014) |
| Imatinib | HEK293 |  | not a Substrate | (Nies et al., 2014) |
| Levorphanol | HEK293 | 0.05-0.1 | Uptake ratio between 1.5 and 2 | (Meyer et al., 2019) |
| Methadone | HEK293 | 1 | Uptake ratio between 1.5 and 2 | (Campbell et al., 2015) |
| Metoclopramide | HEK293 | 2.5 | Uptake ratio between 1.5 and 2 | (Hendrickx et al., 2013) |
| O-desmethyl tramadol | HEK293 | 1 | Uptake ratio between 1.5 and 2 | (Seitz et al., 2015) |
| Ondansetron | HEK293 |  | Uptake ratio between 1.5 and 2 | (Morse et al., 2020) |
| Paraquat | HEK293 | 4.5 | Uptake ratio between 1.5 and 2 | (Chen et al., 2007) |
| Perphenazine | HEK293 | 2.5 | Uptake ratio between 1.5 and 2 | (Hendrickx et al., 2013) |
| Pitavastatin | HEK293 | 1 | Uptake ratio between 1.5 and 2 | (Bi et al., 2019) |
| Rosuvastatin | HEK293 | 1 | Uptake ratio between 1.5 and 2 | (Bi et al., 2019) |
| Sorafenib | *Xenopus* oocytes | 50 | Uptake ratio between 1.5 and 2 | (Herraez et al., 2013) |
| Sorafenib | *Xenopus* oocytes |  | not a Substrate | (Chen et al., 2020) |
| Sorafenib | HEK293 |  | not a Substrate | (Chen et al., 2020) |
| Tapentadol | HEK293 | 0.1-0.5 | Uptake ratio between 1.5 and 2 | (Meyer et al., 2019) |
| Triamterene | HEK293 | 2.5 | Uptake ratio between 1.5 and 2 | (Hendrickx et al., 2013) |
| Trimethoprim | HEK293 | 2.5 | Uptake ratio between 1.5 and 2 | (Hendrickx et al., 2013) |
| Tropisetron | HEK293 |  | Uptake ratio between 1.5 and 2 | (Morse et al., 2020) |
| Varenicline | HEK293 | 2.5 | Uptake ratio between 1.5 and 2 | (Hendrickx et al., 2013) |

Andreev, E., Brosseau, N., Carmona, E., Mes-Masson, A.M., and Ramotar, D. (2016). The human organic cation transporter OCT1 mediates high affinity uptake of the anticancer drug daunorubicin. *Sci Rep* 6**,** 20508. 10.1038/srep20508

Bi, Y.A., Costales, C., Mathialagan, S., West, M., Eatemadpour, S., Lazzaro, S., et al. (2019). Quantitative contribution of six major transporters to the hepatic uptake of drugs: "SLC-Phenotyping" using primary human hepatocytes. *J Pharmacol Exp Ther* 370**,** 72-83. 10.1124/jpet.119.257600

Blanc Mettral, J., Faller, N., Cruchon, S., Sottas, L., Buclin, T., Schild, L., et al. (2019). Imatinib uptake into cells is not mediated by organic cation transporters OCT1, OCT2, or OCT3, but is influenced by extracellular pH. *Drug Metab Lett* 13**,** 102-110. 10.2174/1872312813666190207150207

Campbell, S.D., Gadel, S., Friedel, C., Crafford, A., Regina, K.J., and Kharasch, E.D. (2015). Influence of HIV antiretrovirals on methadone N-demethylation and transport. *Biochem Pharmacol* 95**,** 115-125. 10.1016/j.bcp.2015.03.007

Chen, M., Neul, C., Schaeffeler, E., Frisch, F., Winter, S., Schwab, M., et al. (2020). Sorafenib activity and disposition in liver cancer does not depend on organic cation transporter 1. *Clin Pharmacol Ther* 107**,** 227-237. 10.1002/cpt.1588

Chen, Y., Zhang, S., Sorani, M., and Giacomini, K.M. (2007). Transport of paraquat by human organic cation transporters and multidrug and toxic compound extrusion family. *J Pharmacol Exp Ther* 322**,** 695-700. 10.1124/jpet.107.123554

Ciarimboli, G., Lancaster, C.S., Schlatter, E., Franke, R.M., Sprowl, J.A., Pavenstädt, H., et al. (2012). Proximal tubular secretion of creatinine by organic cation transporter OCT2 in cancer patients. *Clin Cancer Res* 18**,** 1101-1108. 10.1158/1078-0432.CCR-11-2503

Gründemann, D., Hahne, C., Berkels, R., and Schömig, E. (2003). Agmatine is efficiently transported by non-neuronal monoamine transporters extraneuronal monoamine transporter (EMT) and organic cation transporter 2 (OCT2). *J Pharmacol Exp Ther* 304**,** 810-817. 10.1124/jpet.102.044404

Hendrickx, R., Johansson, J.G., Lohmann, C., Jenvert, R.M., Blomgren, A., Börjesson, L., et al. (2013). Identification of novel substrates and structure-activity relationship of cellular uptake mediated by human organic cation transporters 1 and 2. *J Med Chem* 56**,** 7232-7242. 10.1021/jm400966v

Herraez, E., Lozano, E., Macias, R.I., Vaquero, J., Bujanda, L., Banales, J.M., et al. (2013). Expression of SLC22A1 variants may affect the response of hepatocellular carcinoma and cholangiocarcinoma to sorafenib. *Hepatology* 58**,** 1065-1073. 10.1002/hep.26425

Imamura, Y., Murayama, N., Okudaira, N., Kurihara, A., Okazaki, O., Izumi, T., et al. (2011). Prediction of fluoroquinolone-induced elevation in serum creatinine levels: a case of drug-endogenous substance interaction involving the inhibition of renal secretion. *Clin Pharmacol Ther* 89**,** 81-88. 10.1038/clpt.2010.232

Kimura, N., Masuda, S., Katsura, T., and Inui, K. (2009). Transport of guanidine compounds by human organic cation transporters, hOCT1 and hOCT2. *Biochem Pharmacol* 77**,** 1429-1436. 10.1016/j.bcp.2009.01.010

Lepist, E.I., Zhang, X., Hao, J., Huang, J., Kosaka, A., Birkus, G., et al. (2014). Contribution of the organic anion transporter OAT2 to the renal active tubular secretion of creatinine and mechanism for serum creatinine elevations caused by cobicistat. *Kidney Int* 86**,** 350-357. 10.1038/ki.2014.66

Meyer, M.J., Neumann, V.E., Friesacher, H.R., Zdrazil, B., Brockmöller, J., and Tzvetkov, M.V. (2019). Opioids as substrates and inhibitors of the genetically highly variable organic cation transporter OCT1. *J Med Chem* 62**,** 9890-9905. 10.1021/acs.jmedchem.9b01301

Morse, B.L., Kolur, A., Hudson, L.R., Hogan, A.T., Chen, L.H., Brackman, R.M., et al. (2020). Pharmacokinetics of organic cation transporter 1 (OCT1) substrates in Oct1/2 knockout mice and species difference in hepatic OCT1-mediated uptake. *Drug Metab Dispos* 48**,** 93-105. 10.1124/dmd.119.088781

Nies, A.T., Schaeffeler, E., Van Der Kuip, H., Cascorbi, I., Bruhn, O., Kneba, M., et al. (2014). Cellular uptake of imatinib into leukemic cells is independent of human organic cation transporter 1 (OCT1). *Clin Cancer Res* 20**,** 985-994. 10.1158/1078-0432.CCR-13-1999

Obianom, O.N., Coutinho, A.L., Yang, W., Yang, H., Xue, F., and Shu, Y. (2017). Incorporation of a biguanide scaffold enhances drug uptake by organic cation transporters 1 and 2. *Mol Pharm* 14**,** 2726-2739. 10.1021/acs.molpharmaceut.7b00285

Seitz, T., Stalmann, R., Dalila, N., Chen, J., Pojar, S., Dos Santos Pereira, J.N., et al. (2015). Global genetic analyses reveal strong inter-ethnic variability in the loss of activity of the organic cation transporter OCT1. *Genome Med* 7**,** 56. 10.1186/s13073-015-0172-0

Urakami, Y., Kimura, N., Okuda, M., and Inui, K. (2004). Creatinine transport by basolateral organic cation transporter hOCT2 in the human kidney. *Pharm Res* 21**,** 976-981. 10.1023/b:pham.0000029286.45788.ad

Wang, L., Giannoudis, A., Lane, S., Williamson, P., Pirmohamed, M., and Clark, R.E. (2008). Expression of the uptake drug transporter hOCT1 is an important clinical determinant of the response to imatinib in chronic myeloid leukemia. *Clin Pharmacol Ther* 83**,** 258-264. 10.1038/sj.clpt.6100268
