# Supplementary material for: Transport of Drugs and Endogenous Compounds Mediated by Human OCT1: Studies in Single- and Double-Transfected Cell Models
Source: Front Pharmacol. 2021 Apr 22;12:662535. doi: 10.3389/fphar.2021.662535 (PMC8100673; doi:10.3389/fphar.2021.662535)
Supplement: Supplementary file 2 [file table2.docx]

Table S2 Inhibitors of the OCT1-mediated transport in single-transfected cell lines

| **Drug/ compound** | **Cell model** | **Substrate** | **IC_50_ [µM]** | **>Control** | **Reference** |
| --- | --- | --- | --- | --- | --- |
| [H_2_L^Et^]Cl | HEK293 | MPP^+^ | 168.6±26.2 |  | (Milunović et al., 2020) |
| [H_2_L^Et^]Cl copper complex | HEK293 | MPP^+^ | 32.2±2.9 |  | (Milunović et al., 2020) |
| [H_2_L^H^]Cl | HEK293 | MPP^+^ | 740.2±194.8 |  | (Milunović et al., 2020) |
| [H_2_L^H^]Cl copper complex | HEK293 | MPP^+^ | 268.6±31.5 |  | (Milunović et al., 2020) |
| [H_2_L^Me^]Cl | HEK293 | MPP^+^ | 390.8±40.0 |  | (Milunović et al., 2020) |
| [H_2_L^Me^]Cl copper complex | HEK293 | MPP^+^ | 113.4±21.8 |  | (Milunović et al., 2020) |
| [H_2_L^Ph^]Cl | HEK293 | MPP^+^ | 118.9±50.0 |  | (Milunović et al., 2020) |
| [H_2_L^Ph^]Cl copper complex | HEK293 | MPP^+^ | 0.25±0.08 |  | (Milunović et al., 2020) |
| 1-(2-hydroxyethyl)-3-phenylpyridinium | HeLa | TEA | 31.1±2.3 |  | (Bednarczyk et al., 2003) |
| 1-(2-hydroxyethyl)-4-phenylpyridinium | HeLa | TEA | 16.2±1.4 |  | (Bednarczyk et al., 2003) |
| 1-(2-hydroxyethyl)quinolinium | HeLa | TEA | 80.6±3.2 |  | (Bednarczyk et al., 2003) |
| 1-(3-phenylpropyl)-biguanide | HEK293 | MPP^+^ |  | * | (Obianom et al., 2017) |
| 1-(4-phenyl-butyl)-biguanide | HEK293 | MPP^+^ |  | * | (Obianom et al., 2017) |
| 1-(m-phenoxyphenyl)-biguanide | HEK293 | MPP^+^ |  | * | (Obianom et al., 2017) |
| 1-(p-chlorophenethyl)-biguanide | HEK293 | MPP^+^ |  | * | (Obianom et al., 2017) |
| 1-(p-chlorophenyl)-biguanide | HEK293 | MPP^+^ |  | * | (Obianom et al., 2017) |
| 1-(phenyl)methyl-3-phenylpyridinium | HeLa | TEA | 5.5±0.9 |  | (Bednarczyk et al., 2003) |
| 1-(phenyl)methyl-4-phenylpyridinium | HeLa | TEA | 9.3±0.6 |  | (Bednarczyk et al., 2003) |
| 1-(phenyl)methylquinolinium | HeLa | TEA | 14.3±0.7 |  | (Bednarczyk et al., 2003) |
| 1-[p-(p-phenoxy)phenyl]-biguanide | HEK293 | MPP^+^ |  | * | (Obianom et al., 2017) |
| 1-benzyl-1,2,3,4-tetrahydroisoquinoline | HEK293 | ASP^+^ | 82.1 |  | (Chen et al., 2017a) |
| 1-ethyl-3-phenylpyridinium | HeLa | TEA | 28.3±4.5 |  | (Bednarczyk et al., 2003) |
| 1-ethyl-4-phenylpyridinium | HeLa | TEA | 7.1±1.1 |  | (Bednarczyk et al., 2003) |
| 1-ethylquinolinium | HeLa | TEA | 67.6±11.2 |  | (Bednarczyk et al., 2003) |
| 1-methyl-4-phenylpyridinium (MPP^+^) | HeLa | TEA | 15.7±1.15 |  | (Bednarczyk et al., 2003) |
| 1-methyl-4-phenylpyridinium (MPP^+^) | CHO and HEK293 | Ethidium | 390±60 |  | (Lee et al., 2009) |
| 1-methyl-4-phenylpyridinium (MPP^+^) | HEK293 | YM155 | 30.4 |  | (Minematsu et al., 2010) |
| 1-methyl-4-phenylpyridinium (MPP^+^) | HEK293 | Fenoterol |  | * | (Morse et al., 2020) |
| 1-methyl-4-phenylpyridinium (MPP^+^) | HEK293 | Ondansetron |  | * | (Morse et al., 2020) |
| 1-methyl-4-phenylpyridinium (MPP^+^) | HEK293 | Sumatriptan |  | * | (Morse et al., 2020) |
| 1-methyl-4-phenylpyridinium (MPP^+^) | HEK293 | Tropisetron |  | * | (Morse et al., 2020) |
| 1-methyl-4-phenylpyridinium (MPP^+^) | HEK293 | Debrisoquine |  | * | (Neul et al., 2021) |
| 1-methyl-4-phenylpyridinium (MPP^+^) | HEK293 | Sparteine |  | * | (Neul et al., 2021) |
| 1-methyl-4-phenylpyridinium (MPP^+^) | HEK293 | TEA | 23.9 |  | (Umehara et al., 2007) |
| 1-methyl-4-phenylpyridinium (MPP^+^) | HeLa | TEA | 12.3 |  | (Zhang et al., 1998) |
| 2-(2,4-dichlorophenyl)ethyl-biguanide | HEK293 | Metformin |  | * | (Obianom et al., 2017) |
| 2-(2,4-dichlorophenyl)ethyl-biguanide | HEK293 | MPP^+^ |  | * | (Obianom et al., 2017) |
| 2-(4-biphenyl)ethyl-biguanide | HEK293 | MPP^+^ |  | * | (Obianom et al., 2017) |
| 2,2-diphenylethyl-biguanide | HEK293 | MPP^+^ |  | * | (Obianom et al., 2017) |
| 2,3-dihydro-1H-inden-2-yl-biguanide | HEK293 | MPP^+^ |  | * | (Obianom et al., 2017) |
| 2,9-dimethyl-1,4,9-dihydro-3H-β-carbolin-ium | HEK293 | ASP^+^ | 40.4±2.4 |  | (Wagner et al., 2017) |
| 2-amino-3,4-dimethyl-3H imidazo[4,5-f]quinoline | HEK293 | ASP^+^ |  | * | (Sayyed et al., 2019) |
| 2-amino-3,7,8 trimethylimidazo[4,5-f]quinoxaline | HEK293 | ASP^+^ |  | * | (Sayyed et al., 2019) |
| 2-methoxyestradiol | HEK293 | ASP^+^ |  | * | (Ahlin et al., 2008) |
| 3,4-methylenedioxymethamphetamine | HEK293 | MPP^+^ | 24.2±9.20 |  | (Amphoux et al., 2006) |
| 3-amino-1,4-dimethyl-5H-pyrido [4,3-b]indole | HEK293 | ASP^+^ | 2.8±0.5 |  | (Sayyed et al., 2019) |
| 3-amino-1-methyl-5H-pyrido[4,3-b]-indole | HEK293 | ASP^+^ | 3.6±0.6 |  | (Sayyed et al., 2019) |
| 3-heptylbenzyl-biguanide | HEK293 | MPP^+^ |  | * | (Obianom et al., 2017) |
| 3-methoxymorphinan |  | ASP^+^ | 10.29±2.1 | PAMPA | (Meyer et al., 2019) |
| 4-aminobiphenyl | HEK293 | TEA |  | * | (Sayyed et al., 2016) |
| Abacavir | HEK293 | Lamivudine |  | * | (Arimany-Nardi et al., 2016) |
| Abacavir | CHO | MPP^+^ | 0.072±0.033 nM |  | (Minuesa et al., 2009) |
| Abacavir | KCL22 | TEA |  | * | (Moss et al., 2015) |
| Acebutol | MDCK | Atenolol |  | * | (Mimura et al., 2015) |
| Acebutolol | HeLa | TEA | 95.8 |  | (Zhang et al., 1998) |
| Acetylcholine | *Xenopus* oocytes | MPP^+^ | 580±110 |  | (Lips et al., 2005) |
| Aciclovir | S2 | TEA |  | * | (Takeda et al., 2002) |
| Afatinib | HEK293 | MPP^+^ | 80±8 |  | (Johnston et al., 2014) |
| Aflatoxin B1 | S2 | TEA | 64.4 |  | (Tachampa et al., 2008) |
| Agmatine | HEK293 | MPP^+^ | 24000 |  | (Gründemann et al., 2003) |
| Alfuzosin | HEK293 | ASP^+^ | 14.87 |  | (Chen et al., 2017a) |
| Allethrin | HEK293 | Dopamine |  | * | (Chedik et al., 2017) |
| Allethrin | HEK293 | TEA | 2.6 |  | (Chedik et al., 2017) |
| Amantadine | HEK293 | MPP^+^ | 236±55.7 |  | (Amphoux et al., 2006) |
| Amantadine | HeLa | TEA | 18.4±2.42 |  | (Bednarczyk et al., 2003) |
| Amantadine | HEK293 | YM155 | 39.6 |  | (Minematsu et al., 2010) |
| Amantadine | HeLa | TEA |  | * | (Zhang et al., 1998) |
| Amiloride | HEK293 | ASP^+^ | 57 |  | (Ahlin et al., 2008) |
| Amitriptyline | HEK293 | Rhodamine 123 | 58.1±20.4 |  | (Jouan et al., 2014) |
| Amitriptyline | HEK293 | Sumatriptan | 3.47±0.08 |  | (Matthaei et al., 2016) |
| Amitriptyline | HEK293 | Morphine | 4.4±1.6 |  | (Tzvetkov et al., 2013) |
| Amitriptyline | HEK293 | Morphine | 1.89±0.77 |  | (Zhu et al., 2018) |
| Amitriptyline | HEK293 | ASP^+^ | 16.9 |  | (Ahlin et al., 2008) |
| Amprenavir | HEK293 | ASP^+^ | 40.4±7.3 |  | (Duan et al., 2015) |
| Amprenavir | HEK293 | Serotonin |  | * | (Duan et al., 2015) |
| Amprenavir | KCL22 | TEA |  | * | (Moss et al., 2015) |
| Amsacrine | HEK293 | ASP^+^ | 5.0 |  | (Ahlin et al., 2008) |
| Androstene-dione | HEK293 | ASP^+^ |  | * | (Ahlin et al., 2008) |
| Anisodine | HEK293 | MPP^+^ | 12.9±0.9 |  | (Chen et al., 2019) |
| Apomorphine | HEK293 | ASP^+^ | 20.8 |  | (Ahlin et al., 2008) |
| Artemisinin | MDCK | MPP^+^ | 4.2±5.5 |  | (Hubeny et al., 2016) |
| Atropine | HEK293 | ASP^+^ | 12.2 |  | (Ahlin et al., 2008) |
| Atropine | HEK293 | MPP^+^ | 3.1±0.6 |  | (Chen et al., 2017b) |
| Atropine | HEK293 | MPP^+^ | 1.2±0.2 |  | (Müller et al., 2005) |
| Azidothymidine | CHO | MPP^+^ | 0.155±0.111 nM |  | (Minuesa et al., 2009) |
| Baicalein | MDCK | Atenolol |  | * | (Mimura et al., 2015) |
| Baicalin | MDCK | Atenolol |  | * | (Mimura et al., 2015) |
| Bendamustine | CHO | MPP^+^ |  | * | (Gupta et al., 2012) |
| Bendamustine | HEK293 | ASP^+^ | >200 |  | (Huber et al., 2015) |
| Bendamustine ester 2 | HEK293 | ASP^+^ | 11.3±1.9 |  | (Huber et al., 2015) |
| Bendamustine ester 4 | HEK293 | ASP^+^ | 10.1±2.5 |  | (Huber et al., 2015) |
| Bendamustine ester 5 | HEK293 | ASP^+^ | 0.35±0.03 |  | (Huber et al., 2015) |
| Berberine | HEK293 | MPP^+^ |  | * | (Boxberger et al., 2018) |
| Berberine | HEK293 | Serotonin |  | * | (Boxberger et al., 2018) |
| Berberine | HEK293 | Metformin | 7.28 |  | (Kwon et al., 2015) |
| Berberine | MDCK | MPP^+^ |  | * | (Nies et al., 2008) |
| Berberine | MDCK | DAPI |  | * | (Yasujima et al., 2011) |
| Berberrubine | MDCK | MPP^+^ | 1.26±0.19 |  | (Li et al., 2016) |
| Bilastine | CHO | TEA | ≥300 | * | (Lucero et al., 2012) |
| BIM-II | HEK293 | TEA |  | * | (Mayati et al., 2015) |
| BIM-III | HEK293 | TEA |  | * | (Mayati et al., 2015) |
| BIM-VI | HEK293 | TEA |  | * | (Mayati et al., 2015) |
| BIM-VII | HEK293 | TEA |  | * | (Mayati et al., 2015) |
| Bisphenol A | HEK293 | TEA | 39.0±1.2 |  | (Bruyère et al., 2017) |
| Bisphenol F | HEK294 | TEA |  | * | (Bruyère et al., 2017) |
| Bithionol | HEK293 | ASP^+^ | 2.23 |  | (Chen et al., 2017a) |
| Bromosulfophthalein | HEK293 | Serotonin |  | * | (Boxberger et al., 2018) |
| Bromosulfophthalein | HEK293 | TEA |  | * | (Boxberger et al., 2018) |
| Bucindolol | HEK293 | ASP^+^ | 27.2 |  | (Ahlin et al., 2008) |
| Bucindolol | HEK293 | ASP^+^ | 15.1±1.24 |  | (Ahlin et al., 2011) |
| Butylguanidine | HEK293 | TEA | 210±20 |  | (Kimura et al., 2009) |
| Butylscopolamine | HEK293 | MPP^+^ | 28.4±1.8 |  | (Chen et al., 2017b) |
| Butylscopolamine | HEK293 | MPP^+^ | 16±6 |  | (Müller et al., 2005) |
| Cabotegravir | HEK293 | Metformin | >30 |  | (Reese et al., 2016) |
| Camptothecin derivative 11 | HEK293 | MPP^+^ | 5.6±1.2 |  | (Zheng et al., 2016) |
| Camylofine | HEK293 | ASP^+^ | 9.12 |  | (Chen et al., 2017a) |
| Canagliflozin | *Xenopus* oocytes | TEA | 5.2 |  | (Mamidi et al., 2017) |
| Carbamazepin | MDCK | Atenolol |  | * | (Mimura et al., 2015) |
| Carbetapentane | HEK293 | ASP^+^ | 1.55 |  | (Chen et al., 2017a) |
| Carvedilol | MDCK | MPP^+^ | 1.4 |  | (Grube et al., 2011) |
| Carvedilol | HEK293 | ASP^+^ | 3.34 |  | (Chen et al., 2017a) |
| Cediranib | HEK293 | MPP^+^ | 54±2 |  | (Johnston et al., 2014) |
| Cepharanthine | KCL22 | TEA |  | * | (Moss et al., 2015) |
| Chelerythrine | HEK293 | MPP^+^ | 13.60±2.81 |  | (Shams et al., 2018) |
| Chloroquine | MDCK | MPP^+^ | 12.8±9.1 |  | (Hubeny et al., 2016) |
| Chlorpromazine | HEK293 | ASP^+^ | 27 |  | (Ahlin et al., 2008) |
| Chlorpromazine | HEK293 | ASP^+^ | 52.3±1.23 |  | (Ahlin et al., 2011) |
| Chlorpromazine | HeLa | TEA | 4.3±0.16 |  | (Bednarczyk et al., 2003) |
| Chlorprotixen | HEK293 | ASP^+^ | 77.8 |  | (Ahlin et al., 2008) |
| Choline | HeLa | TEA | 3540±24.0 |  | (Bednarczyk et al., 2003) |
| Cigarette smoke condensate | HEK293 | TEA | 12.5±1.5 µg/mL |  | (Sayyed et al., 2016) |
| Cimetidine | CHO | ASP^+^ | 95 |  | (Ciarimboli et al., 2004) |
| Cimetidine | HEK293 | ASP^+^ | >100 |  | (Kido et al., 2011) |
| Cimetidine | CHO and HEK293 | Ethidium | 2830±720 |  | (Lee et al., 2009) |
| Cimetidine | HEK293 | YM155 | 149 |  | (Minematsu et al., 2010) |
| Cimetidine | HEK293 | Nadolol |  | * | (Misaka et al., 2016) |
| Cimetidine | HEK293 | para-Aminosalicylic acid | 107.5±19.5 |  | (Parvez et al., 2017) |
| Cimetidine | HEK293 | MPP^+^ | 1010 |  | (Umehara et al., 2007) |
| Cimetidine | HEK293 | TEA | 571 |  | (Umehara et al., 2007) |
| Cimetidine | HeLa | TEA | 166 |  | (Zhang et al., 1998) |
| Ciprofloxacin | HEK293 | TEA |  | * | (Mulgaonkar et al., 2013) |
| Cisplatin | HEK293 | TEA |  | * | (Yonezawa et al., 2006) |
| Citalopram | HEK293 | ASP^+^ | 18.8 |  | (Ahlin et al., 2008) |
| Citreoveridine | S2 | TEA | 6.63 |  | (Tachampa et al., 2008) |
| Clarithromycin | HEK293 | TEA | >50 |  | (Vermeer et al., 2016) |
| Clemastine | HEK293 | ASP^+^ | 4.9 |  | (Ahlin et al., 2008) |
| Clomipramin | HEK293 | Morphine | 4.6±0.6 |  | (Tzvetkov et al., 2013) |
| Clomipramine | HEK293 | ASP^+^ | 19.3 |  | (Ahlin et al., 2008) |
| Clonidine | HEK293 | ASP^+^ | 22.6 |  | (Ahlin et al., 2008) |
| Clonidine | HeLa | TEA | 0.71±0.08 |  | (Bednarczyk et al., 2003) |
| Clonidine | HEK293 | ASP^+^ | 18.98 |  | (Chen et al., 2017a) |
| Clonidine | HEK293 | Metformin |  | * | (Floerl et al., 2020) |
| Clonidine | HEK293 | MPP^+^ |  | * | (Floerl et al., 2020) |
| Clonidine | HEK293 | MPP^+^ | 6.5±1.3 |  | (Müller et al., 2005) |
| Clonidine | MDCK | DAPI |  | * | (Yasujima et al., 2011) |
| Clonidine | HeLa | TEA | 0.55 |  | (Zhang et al., 1998) |
| Cloperastine | HEK293 | ASP^+^ | 14.89 |  | (Chen et al., 2017a) |
| Clopidogrel | MDCK | Amantadine | 4.15±1.2 |  | (Li et al., 2014a) |
| Clopidogrel | MDCK | Lamivudine | 2.95±0.54 |  | (Li et al., 2014a) |
| Clopidogrel | MDCK | Metformin | 0.307±0.03 |  | (Li et al., 2014a) |
| Clopidogrel | MDCK | MPP^+^ | 0.79±0.46 |  | (Li et al., 2014a) |
| Clopidogrel carboxylate | MDCK | Lamivudine | 1.97±0.55 |  | (Li et al., 2014a) |
| Clopidogrel carboxylate | MDCK | Metformin | 6.25±2.7 |  | (Li et al., 2014a) |
| Clopidogrel carboxylate | MDCK | MPP^+^ | 14±2.3 |  | (Li et al., 2014a) |
| Closantel | HEK293 | ASP^+^ | 3.00 |  | (Chen et al., 2017a) |
| Clotrimazole | HEK293 | ASP^+^ |  | * | (Ahlin et al., 2008) |
| Clotrimazole | HEK293 | ASP^+^ | 11.97 |  | (Chen et al., 2017a) |
| Cocaine | HEK293 | MPP^+^ | 85.0±22 |  | (Amphoux et al., 2006) |
| Codeine |  | ASP^+^ | 235.50±29.4 | PAMPA | (Meyer et al., 2019) |
| Codeine | HEK293 | Morphine | 10.9±0.8 |  | (Tzvetkov et al., 2013) |
| Codeine | HEK293 | MPP^+^ | 10.5±1.3 |  | (Tzvetkov et al., 2013) |
| Coptisine | MDCK | MPP^+^ | 0.931±0.09 |  | (Li et al., 2016) |
| Corticosterone | HEK293 | MPP^+^ | 21.7±2.44 |  | (Hayer-Zillgen et al., 2002) |
| Corticosterone | HEK293 | YM155 | 8.89 |  | (Minematsu et al., 2010) |
| Corticosterone | MDCK | DAPI |  | * | (Yasujima et al., 2011) |
| Corticosterone | MDCK | TEA |  | * | (Yasujima et al., 2011) |
| Corticosterone | HeLa | TEA | 7.02 |  | (Zhang et al., 1998) |
| Cortisone | MDCK | DAPI |  | * | (Yasujima et al., 2011) |
| Corydaline | MDCK | MPP^+^ | 2.61±1.3 |  | (Li et al., 2016) |
| Cremophor EL | MDCK | MPP^+^ | 0.019 % (w/v) |  | (Otter et al., 2017) |
| Crizotinib | HEK293 | *m*IBG | 40.3±5.5 |  | (Lopez Quinones et al., 2020) |
| Crystal violet | HeLa | TEA | 7.7±1.94 |  | (Bednarczyk et al., 2003) |
| Cyclohexylamine | HeLa | TEA | 544±19.0 |  | (Bednarczyk et al., 2003) |
| Cyclosporine A | HEK293 | Cycloguanil | 0.32±0.07 |  | (Panfen et al., 2019) |
| Cyclosporine A | HEK293 | Cycloguanil | 0.15±0.04 |  | (Panfen et al., 2019) |
| Cyclosporine A | HEK293 | Fenoterol | >20 |  | (Panfen et al., 2019) |
| Cyclosporine A | HEK293 | Fenoterol | 2.4±0.9 |  | (Panfen et al., 2019) |
| Cyclosporine A | HEK293 | Metformin | 21.6±4.5 |  | (Panfen et al., 2019) |
| Cyclosporine A | HEK293 | Metformin | 0.43±0.12 |  | (Panfen et al., 2019) |
| Cyclosporine A | HEK293 | MPP^+^ | >20 |  | (Panfen et al., 2019) |
| Cyclosporine A | HEK293 | MPP^+^ | 16.1±6.0 |  | (Panfen et al., 2019) |
| Cyclosporine A | HEK293 | Ranitidine | >20 |  | (Panfen et al., 2019) |
| Cyclosporine A | HEK293 | Ranitidine | 4.6±0.6 |  | (Panfen et al., 2019) |
| Cyclosporine A | HEK293 | Sumatriptan | 2.5±1.2 |  | (Panfen et al., 2019) |
| Cyclosporine A | HEK293 | Sumatriptan | 0.77±0.18 |  | (Panfen et al., 2019) |
| Cyclosporine A | HEK293 | TEA | >20 |  | (Panfen et al., 2019) |
| Cyclosporine A | HEK293 | TEA | 12.1±1.7 |  | (Panfen et al., 2019) |
| Cyproterone | HEK293 | ASP^+^ |  | * | (Ahlin et al., 2008) |
| D-Amphetamine | HEK293 | MPP^+^ | 202±68 |  | (Amphoux et al., 2006) |
| D-Amphetamine | HEK293 | Metformin | 96.7±37 |  | (Wagner et al., 2017) |
| Darunavir | KCL22 | TEA | 15.9 |  | (Moss et al., 2015) |
| Dasatinib | HEK293 | Metformin | 1.07±0.21 |  | (Minematsu and Giacomini, 2011) |
| Debrisoquine | HEK293 | TEA | 2.1±1.0 |  | (Neul et al., 2021) |
| Debrisoquine | HEK293 | MPP^+^ | 6,2±0,8 |  | (Saadatmand et al., 2012) |
| Decynium 22 | HEK293 | MPP^+^ | 0.98±0.31 |  | (Hayer-Zillgen et al., 2002) |
| Decynium 22 | *Xenopus* oocytes | MPP^+^ | 4.7±1.28 |  | (Zhang et al., 1997) |
| Decynium 22 | HeLa | TEA | 2.73±1.24 |  | (Zhang et al., 1998) |
| Denopamine | HEK293 | ASP^+^ | 46.9 |  | (Ahlin et al., 2008) |
| Desipramine | HEK293 | ASP^+^ | 56.8 |  | (Ahlin et al., 2008) |
| Desipramine | HEK293 | ASP^+^ | 43.9±1.44 |  | (Ahlin et al., 2011) |
| Desipramine | HEK293 | ASP^+^ | 9.18 |  | (Chen et al., 2017a) |
| Desipramine | MDCK | DAPI |  | * | (Yasujima et al., 2011) |
| Desipramine | MDCK | TEA |  | * | (Yasujima et al., 2011) |
| Desipramine | HeLa | TEA | 5.36 |  | (Zhang et al., 1998) |
| Dextromethorphan | HEK293 | ASP^+^ | 10.45 |  | (Chen et al., 2017a) |
| Dextromethorphan |  | ASP^+^ | 15.15±2.5 | PAMPA | (Meyer et al., 2019) |
| Dextrorphan |  | ASP^+^ | 6.40±1.6 | PAMPA | (Meyer et al., 2019) |
| Dichlorophene | HEK293 | ASP^+^ | 8.41 |  | (Chen et al., 2017a) |
| Diclofenac | S2 | TEA |  | * | (Khamdang et al., 2002) |
| Diltiazem | HEK293 | ASP^+^ | 12.4 |  | (Ahlin et al., 2008) |
| Diltiazem | MDCK | MPP^+^ | 1.7 |  | (Grube et al., 2011) |
| Diltiazem | HEK293 | MPP^+^ | 15.8 |  | (Umehara et al., 2008) |
| Diltiazem | MDCK | DAPI |  | * | (Yasujima et al., 2011) |
| Dimethylguanidine | HEK293 | TEA | 540±90 |  | (Kimura et al., 2009) |
| Diphenhydramine | HEK293 | Serotonin | 4.1±1.4 |  | (Boxberger et al., 2014) |
| Diphenhydramine | HEK293 | MPP^+^ |  | * | (Boxberger et al., 2018) |
| Diphenhydramine | MDCK | DAPI |  | * | (Yasujima et al., 2011) |
| Diphenhydramine | MDCK | TEA |  | * | (Yasujima et al., 2011) |
| Diphenhydramine | HEK293 | MPP^+^ | 3.4±0.6 |  | (Müller et al., 2005) |
| Dipyramidole | HEK293 | ASP^+^ | 81 |  | (Kido et al., 2011) |
| Disopyramide | HEK293 | ASP^+^ | 81.7 |  | (Ahlin et al., 2008) |
| Disopyramide | HEK293 | ASP^+^ | >100 |  | (Kido et al., 2011) |
| Disopyramide | MDCK | DAPI |  | * | (Yasujima et al., 2011) |
| Disopyramide | HeLa | TEA |  | * | (Zhang et al., 1998) |
| Dobutamine | HEK293 | ASP^+^ | 4.17 |  | (Chen et al., 2017a) |
| Domperidone | HEK293 | Sumatriptan | 32.8±7.4 |  | (Matthaei et al., 2016) |
| Dopamine | HeLa | TEA | 487.2±43.3 |  | (Bednarczyk et al., 2003) |
| Dopamine | MDCK | DAPI |  | * | (Yasujima et al., 2011) |
| Dopamine | HeLa | TEA |  | * | (Zhang et al., 1998) |
| Doxazosin | HEK293 | ASP^+^ |  | * | (Ahlin et al., 2008) |
| Doxazosin | HEK293 | ASP^+^ | 14.8±1.22 |  | (Ahlin et al., 2011) |
| Doxepin | HEK293 | ASP^+^ | 11.19 |  | (Chen et al., 2017a) |
| Efavirenz | HEK293 | Lamivudine |  | * | (Arimany-Nardi et al., 2016) |
| Efavirenz | MDCK | Metformin | 2.30 |  | (Ceckova et al., 2018) |
| Efavirenz | KCL22 | TEA | 7.4 |  | (Moss et al., 2015) |
| Elacridar | HEK293 | Metformin |  | * | (Floerl et al., 2020) |
| Elacridar | HEK293 | MPP^+^ |  | * | (Floerl et al., 2020) |
| Eletriptan | HEK293 | MPP^+^ | 6.81 |  | (Matthaei et al., 2016) |
| Emtricitabine | CHO | MPP^+^ | 0.02±0.006 nM |  | (Minuesa et al., 2009) |
| Epiberberine | MDCK | MPP^+^ | 1.31±0.35 |  | (Li et al., 2016) |
| Epigallocatechine gallate | HEK293 | Metformin |  | * | (Knop et al., 2015) |
| Erlotinib | HEK293 | MPP^+^ | 0.16 |  | (Bi et al., 2019) |
| Erlotinib | HEK293 | ASP^+^ | 16.24 |  | (Chen et al., 2017a) |
| Erlotinib | HEK293 | MPP^+^ | 86±4 |  | (Johnston et al., 2014) |
| Erlotinib | HEK293 | Metformin | 0.356±0.041 |  | (Minematsu and Giacomini, 2011) |
| Etavirine | KCL22 | TEA |  | * | (Moss et al., 2015) |
| Ethambutol | HEK293 | MPP^+^ | >100 |  | (Parvez et al., 2016) |
| Ethidium |  | MPP^+^ |  | * | (Lee et al., 2009) |
| Ethopropazine | HEK293 | ASP^+^ | 20.46 |  | (Chen et al., 2017a) |
| Etilefrine | HEK293 | MPP^+^ | 447±57 |  | (Müller et al., 2005) |
| Etoposide | CHO | MPP^+^ |  | * | (Gupta et al., 2012) |
| Famotidine | *Xenopus* oocytes | MPP^+^ | 28±2 |  | (Bourdet et al., 2005) |
| Famotidine | HEK293 | MPP^+^ |  | * | (Boxberger et al., 2018) |
| Famotidine | HEK293 | Serotonin |  | * | (Boxberger et al., 2018) |
| Famotidine | HEK293 | MPP^+^ | 65.0 |  | (Umehara et al., 2007) |
| Famotidine | HEK293 | TEA | 24.7 |  | (Umehara et al., 2007) |
| Famotidine | HEK293 | ASP^+^ | >300 |  | (Wittwer et al., 2013) |
| Fenamiphos | HEK293 | DAPI | 27.5±1.2 |  | (Chedik et al., 2019) |
| Fenamiphos | HEK293 | Dopamine | 9.2±1.1 |  | (Chedik et al., 2019) |
| Fenitrothion | HEK293 | DAPI |  | * | (Chedik et al., 2019) |
| Fentanyl | HEK293 | ASP^+^ |  | * | (Ahlin et al., 2008) |
| Fentanyl |  | ASP^+^ | 46.17±6.8 | PAMPA | (Meyer et al., 2019) |
| Flecainide | MDCK | MPP^+^ | 2.5 |  | (Grube et al., 2011) |
| Flecainide | HEK293 | MPP^+^ | 41.5 |  | (Umehara et al., 2008) |
| Fleroxacin | HEK293 | TEA |  | * | (Mulgaonkar et al., 2013) |
| Fluoxetine | HEK293 | Serotonin | 6.2±1.2 |  | (Boxberger et al., 2014) |
| Fluoxetine | HEK293 | MPP^+^ |  | * | (Boxberger et al., 2018) |
| Fluoxetine | HEK293 | Morphine | 6.0±0.3 |  | (Tzvetkov et al., 2013) |
| Fluoxetine | HEK293 | Morphine | 9.06±3.24 |  | (Zhu et al., 2018) |
| Flupentixol | HEK293 | ASP^+^ | 89.5 |  | (Ahlin et al., 2008) |
| Fluphenazine | HEK293 | ASP^+^ | 110 |  | (Ahlin et al., 2008) |
| Forctinib | HEK293 | MPP^+^ | 74±4 |  | (Johnston et al., 2014) |
| Formoterol | HEK293 | MPP^+^ | 22.3 |  | (Salomon et al., 2015) |
| Furaminidine | CHO | MPP^+^ | 7.4±0.9 |  | (Ming et al., 2009) |
| Ganciclovir | S2 | TEA |  | * | (Takeda et al., 2002) |
| Gatifloxacin | HEK293 | TEA | 250±18 |  | (Mulgaonkar et al., 2013) |
| Gefitinib | HEK293 | MPP^+^ | 1.1 |  | (Bi et al., 2019) |
| Gefitinib | HEK293 | MPP^+^ | 57±5 |  | (Johnston et al., 2014) |
| Gefitinib | HEK293 | Metformin | 1.07±0.12 |  | (Minematsu and Giacomini, 2011) |
| Glibenclamid | MDCK | Atenolol |  | * | (Mimura et al., 2015) |
| Gliotoxin | S2 | TEA | 584 |  | (Tachampa et al., 2008) |
| Gö 6850 | HEK293 | TEA |  | * | (Mayati et al., 2015) |
| Gö 6983 | HEK293 | TEA |  | * | (Mayati et al., 2015) |
| Green tea extract | HEK293 | Metformin | 1.4% [v/v] |  | (Knop et al., 2015) |
| Griseofulvin | HEK293 | ASP^+^ | 7.3 |  | (Chen et al., 2017a) |
| Guanabenz | HEK293 | ASP^+^ | 4.85 |  | (Chen et al., 2017a) |
| Guanidinosuccinic acid | HEK293 | TEA | 1540±150 |  | (Kimura et al., 2009) |
| Guanidinovaleric acid | HEK293 | TEA | 660±33 |  | (Kimura et al., 2009) |
| Gugglesteron | HEK293 | ASP^+^ |  | * | (Ahlin et al., 2008) |
| Haloperidol | HEK293 | ASP^+^ | 141.9 |  | (Ahlin et al., 2008) |
| Harmaline | HEK293 | ASP^+^ | 27.6±3.8 |  | (Wagner et al., 2017) |
| Harmane | HEK293 | ASP^+^ |  | * | (Sayyed et al., 2019) |
| Harmane | HEK293 | ASP^+^ | 75.5±10.2 |  | (Wagner et al., 2017) |
| Harmine | HEK293 | ASP^+^ | 23.1±2.8 |  | (Wagner et al., 2017) |
| Hesperetin | MDCK | Atenolol |  | * | (Mimura et al., 2015) |
| Hesperidin | MDCK | Atenolol |  | * | (Mimura et al., 2015) |
| Histamine | HeLa | TEA | 3007±27.2 |  | (Bednarczyk et al., 2003) |
| Homatropine | HEK293 | MPP^+^ | 4.0±0.2 |  | (Chen et al., 2017b) |
| Hydrocodone |  | ASP^+^ | 536.96±87.6 | PAMPA | (Meyer et al., 2019) |
| Hydrocortisone | MDCK | DAPI |  | * | (Yasujima et al., 2011) |
| Hydromorphone |  | ASP^+^ | 137.97±19.2 | PAMPA | (Meyer et al., 2019) |
| Hydroxy-itraconazole | HEK293 | TEA | 0.01±0.00 |  | (Vermeer et al., 2016) |
| Hydroxypropyl-β-cyclodextrin | MDCK | MPP^+^ | 25.6 % (w/v) |  | (Otter et al., 2017) |
| Ibuprofen | S2 | TEA |  | * | (Khamdang et al., 2002) |
| Ifosamide | CHO | MPP^+^ |  | * | (Gupta et al., 2012) |
| Imatinib | HEK293 | Serotonin | 10.2±1.2 |  | (Boxberger et al., 2014) |
| Imatinib | HEK293 | Metformin | 1.47±0.09 |  | (Minematsu and Giacomini, 2011) |
| Imatinib | CHO | MPP^+^ | 0.095±0.03 |  | (Nies et al., 2014) |
| Imatinib | HEK293 | ASP^+^ | 107 |  | (Wittwer et al., 2013) |
| Imazalil | HEK293 | Metformin |  | * | (Floerl et al., 2020) |
| Imazalil | HEK293 | MPP^+^ |  | * | (Floerl et al., 2020) |
| Imeglimin | HEK293 | MPP^+^ | 154 |  | (Chevalier et al., 2020) |
| Imipramine | HEK293 | ASP^+^ | 17.1 |  | (Ahlin et al., 2008) |
| Imipramine | HEK293 | ASP^+^ | 7.95 |  | (Chen et al., 2017a) |
| Imipramine | HEK293 | Quercetin |  | * | (Glaeser et al., 2014) |
| Imipramine | HEK293 | ASP^+^ | 37 |  | (Kido et al., 2011) |
| Imipramine | HEK293 | Morphine | 6.2±1.4 |  | (Tzvetkov et al., 2013) |
| Imipramine | HEK293 | Morphine | 4.03±1.62 |  | (Zhu et al., 2018) |
| Imiprothrin | HEK293 | TEA |  | * | (Chedik et al., 2017) |
| Indinavir | HEK293 | ASP^+^ | 33.4±6.8 |  | (Duan et al., 2015) |
| Indinavir | HEK293 | Serotonin |  | * | (Duan et al., 2015) |
| Indinavir | HEK293 | MPP^+^ | 37±6 |  | (Jung et al., 2008) |
| Indinavir | KCL22 | TEA |  | * | (Moss et al., 2015) |
| Indinavir | HEK293 | ASP^+^ | 208 |  | (Wittwer et al., 2013) |
| Indinavir | HeLa | TEA | 61.7±18.4 |  | (Zhang et al., 2000) |
| Indometacin | S2 | TEA |  | * | (Khamdang et al., 2002) |
| Ipratropium | HEK293 | MPP^+^ | 17.4±2.7 |  | (Chen et al., 2017b) |
| Ipratropium | HEK293 | Fenoterol | 25.7±2.2 |  | (Tzvetkov et al., 2018) |
| Irinotecan | CHO | MPP^+^ | 1.71 |  | (Gupta et al., 2012) |
| Irinotecan | HEK293 | *m*IBG | 5.1±2.7 |  | (Lopez Quinones et al., 2020) |
| Irinotecan | HEK293 | Morphine | 1.5±0.3 |  | (Tzvetkov et al., 2013) |
| Irinotecan | HEK293 | ASP^+^ | 20.8 |  | (Wittwer et al., 2013) |
| Irinotecan | HEK293 | Morphine | 1.36±0.43 |  | (Zhu et al., 2018) |
| Itraconazole | HEK293 | TEA | 0.74±0.24 |  | (Vermeer et al., 2016) |
| Jatrorrhizine | MDCK | MPP^+^ | 0.932±0.09 |  | (Li et al., 2016) |
| Kaempferol | MDCK | Atenolol |  | * | (Mimura et al., 2015) |
| Kaempferol | MDCK | DAPI |  | * | (Yasujima et al., 2011) |
| Ketamine | HEK293 | MPP^+^ | 114.5±43.7 |  | (Amphoux et al., 2006) |
| Ketoconazole | HEK293 | ASP^+^ |  | * | (Ahlin et al., 2008) |
| Ketoconazole | HEK293 | ASP^+^ | 7.4±1.21 |  | (Ahlin et al., 2011) |
| Ketoconazole | HEK293 | ASP^+^ | 2.6 |  | (Chen et al., 2017a) |
| Ketoconazole | HEK293 | Metformin |  | * | (Floerl et al., 2020) |
| Ketoconazole | HEK293 | MPP^+^ |  | * | (Floerl et al., 2020) |
| Ketoconazole | HEK293 | TEA | 0.13±0.03 |  | (Vermeer et al., 2016) |
| Keto-itraconazole | HEK293 | TEA | 0.04±0.01 |  | (Vermeer et al., 2016) |
| Ketoprofen | S2 | TEA |  | * | (Khamdang et al., 2002) |
| KN62 | HEK293 | TEA |  | * | (Mayati et al., 2015) |
| Kolliphor P407 | MDCK | MPP^+^ | 1.85 % (w/v) |  | (Otter et al., 2017) |
| Lamivudine | HEK293 | MPP^+^ |  | * | (Boxberger et al., 2018) |
| Lamivudine | HEK293 | Serotonin |  | * | (Boxberger et al., 2018) |
| Lamivudine | HEK293 | MPP^+^ | 17±3 |  | (Jung et al., 2008) |
| Lamivudine | CHO | MPP^+^ | 12.3±0.25 pM (High-Affinity Binding Site) |  | (Minuesa et al., 2009) |
| Lamivudine | CHO | MPP^+^ | 1900±140 (Low-Affinity Binding Site) |  | (Minuesa et al., 2009) |
| Lamotrigin | KCL22 | TEA | 45 |  | (Dickens et al., 2012) |
| Lansoprazole | HEK293 | Metformin | 35.8±5.8 |  | (Nies et al., 2011) |
| Lansoprazole | HEK293 | para-Aminosalicylic acid | 25.2±3.7 |  | (Parvez et al., 2017) |
| Lapatanib | HEK293 | Metformin | >30 |  | (Minematsu and Giacomini, 2011) |
| Lesinurad | MDCK | Metformin | 13.7 |  | (Shen et al., 2016) |
| Levofloxacin | HEK293 | TEA |  | * | (Mulgaonkar et al., 2013) |
| Levofloxacin | HEK293 | Metformin | 22.6±6.3 |  | (Parvez et al., 2016) |
| Levofloxacin | HEK293 | MPP^+^ | 30.3±4.4 |  | (Parvez et al., 2016) |
| Levorphanol |  | ASP^+^ | 8.48±0.6 | PAMPA | (Meyer et al., 2019) |
| Levo-tetrahydropalmatine | MDCK | Dehydrocordaline |  | * | (Chen et al., 2020) |
| Linagliptin | HEK293 | Metformin | 41 |  | (Ishiguro et al., 2013) |
| Linagliptin | HEK293 | TEA | 45 |  | (Ishiguro et al., 2013) |
| Lomefloxacin | HEK293 | TEA |  | * | (Mulgaonkar et al., 2013) |
| Loperamide | HEK293 | ASP^+^ | 23.7 |  | (Ahlin et al., 2008) |
| Lopinavir | HEK293 | ASP^+^ | 174±40.1 |  | (Duan et al., 2015) |
| Lopinavir | KCL22 | TEA |  | * | (Moss et al., 2015) |
| Loratadine | HEK293 | ASP^+^ |  | * | (Ahlin et al., 2008) |
| Malathion | HEK293 | DAPI |  | * | (Chedik et al., 2019) |
| Mefenamic acid | HEK293 | MPP^+^ | 242 |  | (Bi et al., 2019) |
| Mefenamic acid | S2 | TEA |  | * | (Khamdang et al., 2002) |
| Mefloquine | MDCK | MPP^+^ | 6.6±6.5 |  | (Hubeny et al., 2016) |
| Memantine | HEK293 | ASP^+^ | 27.2 |  | (Ahlin et al., 2008) |
| Memantine | HEK293 | MPP^+^ | 3.7±1.2 |  | (Amphoux et al., 2006) |
| Mepensolate | HEK293 | ASP^+^ | 64.9 |  | (Ahlin et al., 2008) |
| Meptazinol |  | ASP^+^ | 18.96±3.1 | PAMPA | (Meyer et al., 2019) |
| Metformin | *Xenopus* oocytes | MPP^+^ | 1231±330 |  | (Bourdet et al., 2005) |
| Metformin | HEK293 | Thiamin | 1400 |  | (Chen et al., 2014) |
| Metformin | *Xenopus* oocytes | Cimetidin | 2010±220 |  | (Dresser et al., 2002) |
| Metformin | HEK293 | Aminoguanidine | 9480±560 |  | (Kimura et al., 2009) |
| Metformin | CHO | MPP^+^ | 3420±770 |  | (Nies et al., 2009) |
| Metformin | HEK293 | para-Aminosalicylic acid | 179.1±33 |  | (Parvez et al., 2017) |
| Metformin | HEK293 | MPP^+^ | 493 |  | (Umehara et al., 2007) |
| Metformin | HEK293 | TEA | 2820 |  | (Umehara et al., 2007) |
| Methamphetamine | HEK293 | Metformin | 21.1±8.8 |  | (Wagner et al., 2017) |
| Methoxyverapamil | HEK293 | ASP^+^ |  | * | (Ahlin et al., 2008) |
| Methyl-parathion | HEK293 | DAPI |  | * | (Chedik et al., 2019) |
| Methylguanidine | HEK293 | TEA | 2360±60 |  | (Kimura et al., 2009) |
| Methylnaltrexone |  | ASP^+^ | 234.39±30.0 | PAMPA | (Meyer et al., 2019) |
| Metoclopramide | HEK293 | ASP^+^ | 94.8 |  | (Ahlin et al., 2008) |
| Metoclopramide | HEK293 | Sumatriptan | 15.9±8.9 |  | (Matthaei et al., 2016) |
| Metoclopramide | MDCK | DAPI |  | * | (Yasujima et al., 2011) |
| Metoprolol | MDCK | MPP^+^ | 52.6 |  | (Grube et al., 2011) |
| Metoprolol | MDCK | Atenolol |  | * | (Mimura et al., 2015) |
| Metoprolol | HEK293 | MPP^+^ | 268 |  | (Umehara et al., 2008) |
| Midazolam | HeLa | TEA | 3.7 |  | (Zhang et al., 1998) |
| Mitoxantrone | CHO | MPP^+^ | 85.2 |  | (Gupta et al., 2012) |
| Mitoxantrone | HEK293 | ASP^+^ | 43.9 |  | (Wittwer et al., 2013) |
| Mitoxantrone | MDCK | ASP^+^ | 6.24 |  | (Ceckova et al., 2016) |
| MK-801 | HEK293 | MPP^+^ | 80.5±48.7 |  | (Amphoux et al., 2006) |
| Monocrotaline | HEK293 | MPP^+^ | 36.8±5.0 |  | (Chen et al., 2019) |
| Monocrotaline | MDCK | MPP^+^ | 5.52±0.56 |  | (Tu et al., 2013) |
| Morphine | HEK293 | ASP^+^ | 28 |  | (Ahlin et al., 2008) |
| Morphine |  | ASP^+^ | 71.79±6.7 | PAMPA | (Meyer et al., 2019) |
| Morphine | HEK293 | MPP^+^ | 4.2±0.6 |  | (Tzvetkov et al., 2013) |
| Moxifloxacin | HEK293 | TEA | 161±19 |  | (Mulgaonkar et al., 2013) |
| Moxifloxacin | HEK293 | Ethambutol |  | * | (te Brake et al., 2016) |
| Moxifloxacin | HEK293 | Metformin |  | * | (te Brake et al., 2016) |
| N-1-Methylnicotinamide | HeLa | TEA | 1035±58.0 |  | (Bednarczyk et al., 2003) |
| N-1-Methylnicotinamide | HEK293 | YM155 | >1000 |  | (Minematsu et al., 2010) |
| N-1-Methylnicotinamide | HeLa | TEA | 7700 |  | (Zhang et al., 1998) |
| Naltrexone |  | ASP^+^ | 157.51±7.5 | PAMPA | (Meyer et al., 2019) |
| Nandrolon | HEK293 | ASP^+^ | 35.1 |  | (Ahlin et al., 2008) |
| Naratriptan | HEK293 | MPP^+^ | 25.3 |  | (Matthaei et al., 2016) |
| N-desalkyl-itraconazole | HEK293 | TEA | >0.2 |  | (Vermeer et al., 2016) |
| N-desmethyl-imatinib | HEK293 | Metformin | 1.76±0.44 |  | (Minematsu and Giacomini, 2011) |
| N-desmethyl-tramadol |  | ASP^+^ | 55.75±9.4 | PAMPA | (Meyer et al., 2019) |
| Nelfinavir | HEK293 | ASP^+^ | 60.5±10.9 |  | (Duan et al., 2015) |
| Nelfinavir | HEK293 | Serotonin |  | * | (Duan et al., 2015) |
| Nelfinavir | HEK293 | MPP^+^ | 7±1 |  | (Jung et al., 2008) |
| Nelfinavir | KCL22 | TEA |  | * | (Moss et al., 2015) |
| Nelfinavir | HeLa | TEA | 21.8±5.4 |  | (Zhang et al., 2000) |
| Neratinib | HEK293 | MPP^+^ | 77±2 |  | (Johnston et al., 2014) |
| Nevirapine | KCL22 | TEA |  | * | (Moss et al., 2015) |
| Nicotine | HeLa | TEA | 53.2±9.07 |  | (Bednarczyk et al., 2003) |
| Nicotine | HEK293 | TEA |  | * | (Sayyed et al., 2016) |
| Nifedipin | MDCK | MPP^+^ | 31.1 |  | (Grube et al., 2011) |
| Nifekalant | HEK293 | ASP^+^ | >300 |  | (Wittwer et al., 2013) |
| Nilotinib | HEK293 | Metformin | 2.92±0.9 |  | (Minematsu and Giacomini, 2011) |
| Nitidine | MDCK | MPP^+^ | 1.09±0.077 |  | (Li et al., 2014b) |
| Nitroprusside | HEK293 | ASP^+^ | 43.84 |  | (Chen et al., 2017a) |
| N-methylquinidine | *Xenopus* oocytes | Tributylmethylammonium |  | * | (van Montfoort et al., 2001) |
| Norepinephrine | MDCK | DAPI |  | * | (Yasujima et al., 2011) |
| Norfentanyl |  | ASP^+^ | 117.74±16.6 | PAMPA | (Meyer et al., 2019) |
| Norfloxacin | HEK293 | TEA |  | * | (Mulgaonkar et al., 2013) |
| Norharmane | HEK293 | ASP^+^ |  | * | (Sayyed et al., 2019) |
| Norharmanium | HEK293 | ASP^+^ | 70.5±6.6 |  | (Wagner et al., 2017) |
| Norlevorphanol |  | ASP^+^ | 24.93±5.1 | PAMPA | (Meyer et al., 2019) |
| Noroxycodone |  | ASP^+^ | 199.82±22.3 | PAMPA | (Meyer et al., 2019) |
| Nortilidine |  | ASP^+^ | 88.76±18.8 | PAMPA | (Meyer et al., 2019) |
| Nuciferine | MDCK | Metformin | 13.4±0.63 |  | (Li et al., 2018) |
| Octreotide | HEK293 | MPP^+^ | 20 |  | (Bi et al., 2019) |
| O-desmethyl erlotinib | HEK293 | Metformin | 3.62±1.07 |  | (Minematsu and Giacomini, 2011) |
| O-desmethyl gefitinib | HEK293 | Metformin | 2.19±0.57 |  | (Minematsu and Giacomini, 2011) |
| O-desmethyl tramadol | HEK293 | ASP^+^ | 172±73 |  | (Tzvetkov et al., 2011) |
| Ofloxacin | HEK293 | TEA |  | * | (Mulgaonkar et al., 2013) |
| Olaparib | HEK293 | MPP^+^ | 37.9 |  | (McCormick and Swaisland, 2017) |
| Omeprazol | HEK293 | Metformin | 15.7±3.8 |  | (Nies et al., 2011) |
| Omeprazol | HEK293 | para-Aminosalicylic acid | 17.1±2.25 |  | (Parvez et al., 2017) |
| Omeprazol | MDCK | DAPI |  | * | (Yasujima et al., 2011) |
| O-methylioprenaline | HEK293 | MPP^+^ | >100 |  | (Hayer-Zillgen et al., 2002) |
| Ondansetron | HEK293 | ASP^+^ | 20.4 |  | (Ahlin et al., 2008) |
| Ondansetron | HEK293 | ASP^+^ | 36.2±1.4 |  | (Ahlin et al., 2011) |
| Ondansetron | HEK293 | ASP^+^ | >100 |  | (Kido et al., 2011) |
| Ondansetron | HEK293 | ASP^+^ | 63±15,6 |  | (Tzvetkov et al., 2012) |
| Ondansetron | HEK293 | Morphine | 1.2±0.2 |  | (Tzvetkov et al., 2013) |
| Ondansetron | HEK293 | ASP^+^ | >300 |  | (Wittwer et al., 2013) |
| Ondansetron | HEK293 | Morphine | 0.27±0.10 |  | (Zhu et al., 2018) |
| Orphenadrine | HEK293 | ASP^+^ | 12.6 |  | (Ahlin et al., 2008) |
| Orphenadrine | HEK293 | ASP^+^ | >100 |  | (Kido et al., 2011) |
| Oxibutynin | HEK293 | ASP^+^ |  | * | (Ahlin et al., 2008) |
| Oxibutynin | HEK293 | MPP^+^ | 20.0±8.84 |  | (Wenge et al., 2011) |
| Oxprenolol | HEK293 | ASP^+^ | 28.6 |  | (Ahlin et al., 2008) |
| Oxprenolol | HEK293 | MPP^+^ | 87.3 |  | (Umehara et al., 2008) |
| Oxycodone |  | ASP^+^ | 2003.78±172.9 | PAMPA | (Meyer et al., 2019) |
| Oxymorphone |  | ASP^+^ | 250.33±35.2 | PAMPA | (Meyer et al., 2019) |
| Paclitaxel | CHO | MPP^+^ | 50.1 |  | (Gupta et al., 2012) |
| Palmatine | MDCK | MPP^+^ | 2.30±0.49 |  | (Li et al., 2016) |
| Pantoprazole | HEK293 | Metformin | 30.8±8.8 |  | (Nies et al., 2011) |
| Pantoprazole | HEK293 | ASP^+^ | >500 |  | (Wittwer et al., 2013) |
| Papaverin | HEK293 | ASP^+^ |  | * | (Ahlin et al., 2008) |
| para-hydroxymethamphetamine | HEK293 | Metformin | 12.0±3.4 |  | (Wagner et al., 2017) |
| Parathion | HEK293 | DAPI |  | * | (Chedik et al., 2019) |
| Pargyline | HEK293 | MPP^+^ | 260 ± 170 |  | (Cheong et al., 2017) |
| Pazopanib | HEK293 | Metformin | 0.253 |  | (Ellawatty et al., 2018) |
| Pefictinib | HEK293 | Metformin | 0.247 |  | (Shibata et al., 2020) |
| Pefloxacin | HEK293 | TEA |  | * | (Mulgaonkar et al., 2013) |
| Pelitinib | HEK293 | MPP^+^ | 76±1 |  | (Johnston et al., 2014) |
| Pentamidine | HEK293 | MPP^+^ | 0.4±1 |  | (Jung et al., 2008) |
| Pentamidine | CHO | MPP^+^ | 16.4±1.7 |  | (Ming et al., 2009) |
| Pentamidine | HEK293 | ASP^+^ | 22.1 |  | (Wittwer et al., 2013) |
| Perphenazine | MDCK | DAPI |  | * | (Yasujima et al., 2011) |
| Pethidine |  | ASP^+^ | 22.25±3.7 | PAMPA | (Meyer et al., 2019) |
| PFE-1 |  |  |  | * | (Luo et al., 2020) |
| PFE-2 |  |  |  | * | (Luo et al., 2020) |
| Phencyclidine | HEK293 | MPP^+^ | 4.40±1.40 |  | (Amphoux et al., 2006) |
| Phenformin | HEK293 | Thiamin | 70 |  | (Chen et al., 2014) |
| Phenformin | *Xenopus* oocytes | Cimetidin | 10±6.9 |  | (Dresser et al., 2002) |
| Phenformin | HEK293 | MPP^+^ |  | * | (Obianom et al., 2017) |
| Phenformin | HEK293 | MPP^+^ |  |  | (Umehara et al., 2007) |
| Phenformin | HEK293 | TEA |  |  | (Umehara et al., 2007) |
| Phenoxybenzamine | HEK293 | ASP^+^ | 15.1 |  | (Ahlin et al., 2008) |
| Phenoxybenzamine | HEK293 | MPP^+^ | 2.72±0.65 |  | (Hayer-Zillgen et al., 2002) |
| Phenylguanidine | HEK293 | TEA | 230±30 |  | (Kimura et al., 2009) |
| Phloretin | MDCK | Atenolol |  | * | (Mimura et al., 2015) |
| Phlorizin | MDCK | Atenolol |  | * | (Mimura et al., 2015) |
| Phosmet | HEK293 | DAPI | 10.4±1.2 |  | (Chedik et al., 2019) |
| Phosmet | HEK293 | Dopamine | 7.1±1.2 |  | (Chedik et al., 2019) |
| Pindolol | HeLa | TEA | 9.7±0.94 |  | (Bednarczyk et al., 2003) |
| Pindolol | MDCK | Atenolol |  | * | (Mimura et al., 2015) |
| Pindolol | HEK293 | MPP^+^ | 39.1 |  | (Umehara et al., 2008) |
| Pindolol | MDCK | DAPI |  | * | (Yasujima et al., 2011) |
| Piperine | HEK293 | MPP^+^ | 400 ± 92 |  | (Cheong et al., 2017) |
| Piroxicam | S2 | TEA |  | * | (Khamdang et al., 2002) |
| Polyethyleneglycol 400 | MDCK | MPP^+^ | 1.81 % (w/v) |  | (Otter et al., 2017) |
| PP2 | HEK293 | TEA |  | * | (Mayati et al., 2015) |
| Prallethrin | HEK293 | TEA |  | * | (Chedik et al., 2017) |
| Prazosin | HEK293 | ASP^+^ | 9.9 |  | (Ahlin et al., 2008) |
| Prazosin | HEK293 | ASP^+^ | 50.9±1.37 |  | (Ahlin et al., 2011) |
| Prazosin | HEK293 | MPP^+^ | 0.55 |  | (Bi et al., 2019) |
| Prazosin | HEK293 | MPP^+^ | 1.84±0.48 |  | (Hayer-Zillgen et al., 2002) |
| Prazosin | HEK293 | Jatrorrhizine | 2.77±0.72 |  | (Liang et al., 2020) |
| Prazosin | HEK293 | YM155 | 1.56 |  | (Minematsu et al., 2010) |
| Prazosin | KCL22 | TEA | 2.3 |  | (Moss et al., 2015) |
| Procainamide | HeLa | TEA | 14.5±0.83 |  | (Bednarczyk et al., 2003) |
| Procainamide | HEK293 | YM155 | 51.3 |  | (Minematsu et al., 2010) |
| Procainamide | HeLa | TEA | 73.9 |  | (Zhang et al., 1998) |
| Prochlorperazine | HEK293 | ASP^+^ | 49.6 |  | (Ahlin et al., 2008) |
| Profenofos | HEK293 | DAPI |  | * | (Chedik et al., 2019) |
| Progesterone | HEK293 | ASP^+^ |  | * | (Ahlin et al., 2008) |
| Progesterone | HEK293 | MPP^+^ | 3.05±1.36 |  | (Hayer-Zillgen et al., 2002) |
| Progesterone | MDCK | DAPI |  | * | (Yasujima et al., 2011) |
| Promazin | HEK293 | ASP^+^ | 17.2 |  | (Ahlin et al., 2008) |
| Promethazine | HEK293 | ASP^+^ | 35.1 |  | (Ahlin et al., 2008) |
| Promethazine | HEK293 | ASP^+^ | 35.1 |  | (Ahlin et al., 2008) |
| Propafenone | HEK293 | ASP^+^ | 11.1 |  | (Ahlin et al., 2008) |
| Propafenone | HEK293 | ASP^+^ | 14.1±1.18 |  | (Ahlin et al., 2011) |
| Propafenone | HEK293 | ASP^+^ | 15.54 |  | (Chen et al., 2017a) |
| Propafenone | MDCK | MPP^+^ | 1.0 |  | (Grube et al., 2011) |
| Propamocarb | HEK293 | TEA | 48.1±1.2 |  | (Gueniche et al., 2020) |
| Propanolol | MDCK | MPP^+^ | 1.3 |  | (Grube et al., 2011) |
| Propantheline | MDCK | DAPI |  | * | (Yasujima et al., 2011) |
| Propetamphos | HEK293 | DAPI |  | * | (Chedik et al., 2019) |
| Propranolol | HEK293 | ASP^+^ | 63.2 |  | (Ahlin et al., 2008) |
| Propranolol | HEK293 | Sumatriptan | 6.27±0.06 |  | (Matthaei et al., 2016) |
| Propranolol | MDCK | Atenolol |  | * | (Mimura et al., 2015) |
| Propranolol | HEK293 | MPP^+^ | 113 |  | (Umehara et al., 2008) |
| Propranolol | MDCK | DAPI |  | * | (Yasujima et al., 2011) |
| Propranolol | MDCK | TEA |  | * | (Yasujima et al., 2011) |
| Propylguanidine | HEK293 | TEA | 360±40 |  | (Kimura et al., 2009) |
| Prostaglandin F2α | S2 | TEA |  | * | (Kimura et al., 2002) |
| Prulifloxacin | HEK293 | TEA | 136±33 |  | (Mulgaonkar et al., 2013) |
| Pyrazinamide | HEK293 | Metformin | 25.8±4.0 |  | (Parvez et al., 2016) |
| Pyrazinamide | HEK293 | MPP^+^ | 36.5±4.0 |  | (Parvez et al., 2016) |
| Pyrimethamine | HEK293 | ASP^+^ | 13.57 |  | (Chen et al., 2017a) |
| Pyrimethamine | MDCK | MPP^+^ | 8.5±1.9 |  | (Hubeny et al., 2016) |
| Pyrimethamine | HEK293 | TEA | 3.8±0.3 |  | (Ito et al., 2010) |
| Pyrimethamine | HEK293 | Metformin | 1.8±0.2 |  | (Panfen et al., 2019) |
| Pyrimethamine | HEK293 | Metformin | 1.3±0.1 |  | (Panfen et al., 2019) |
| Quercetin | MDCK | Atenolol |  | * | (Mimura et al., 2015) |
| Quercetin | MDCK | DAPI |  | * | (Yasujima et al., 2011) |
| Quercetin-3β-D-glucoside | MDCK | Atenolol |  | * | (Mimura et al., 2015) |
| Quetiapin | KCL22 | Lamotrigin | 1,9 |  | (Dickens et al., 2012) |
| Quinidine | HEK293 | ASP^+^ | 113.8 |  | (Ahlin et al., 2008) |
| Quinidine | HEK293 | ASP^+^ | 340±1.62 |  | (Ahlin et al., 2011) |
| Quinidine | HeLa | TEA | 5.4±0.22 |  | (Bednarczyk et al., 2003) |
| Quinidine | HEK293 | MPP^+^ | 6.5 |  | (Bi et al., 2019) |
| Quinidine | *Xenopus* oocytes | MPP^+^ | 6.7±2.0 |  | (Bourdet et al., 2005) |
| Quinidine | HEK293 | Metformin |  | * | (Floerl et al., 2020) |
| Quinidine | HEK293 | MPP^+^ |  | * | (Floerl et al., 2020) |
| Quinidine | MDCK | Berberrubine |  | * | (Li et al., 2016) |
| Quinidine | MDCK | Coptisine |  | * | (Li et al., 2016) |
| Quinidine | MDCK | Epiberberine |  | * | (Li et al., 2016) |
| Quinidine | MDCK | Jatrorrhizine |  | * | (Li et al., 2016) |
| Quinidine | MDCK | MPP^+^ | 3.63±1.5 |  | (Li et al., 2016) |
| Quinidine | MDCK | Atenolol |  | * | (Mimura et al., 2015) |
| Quinidine | HEK293 | YM155 | 7.11 |  | (Minematsu et al., 2010) |
| Quinidine | CHO | MPP^+^ | 5.7±0.9 |  | (Ming et al., 2009) |
| Quinidine | HEK293 | Metformin | 7.7±0.9 |  | (Panfen et al., 2019) |
| Quinidine | HEK293 | Metformin | 4.5±0.7 |  | (Panfen et al., 2019) |
| Quinidine | HEK293 | para-Aminosalicylic acid | 4.84±1.4 |  | (Parvez et al., 2017) |
| Quinidine | HEK293 | MPP^+^ | 16.8 |  | (Umehara et al., 2008) |
| Quinidine | *Xenopus* oocytes | Tributylmethylammonium |  | * | (van Montfoort et al., 2001) |
| Quinidine | HeLa | TEA | 18 |  | (Zhang et al., 1998) |
| Quinine | HEK293 | ASP^+^ | 51.6 |  | (Ahlin et al., 2008) |
| Quinine | HEK293 | MPP^+^ | 9.2 ± 0.4 |  | (Cheong et al., 2017) |
| Quinine | HEK293 | ASP^+^ | 29 |  | (Ciarimboli et al., 2004) |
| Quinine | CHO | ASP^+^ | 45 |  | (Ciarimboli et al., 2004) |
| Quinine | HEK293 | MPP^+^ |  | * | (Floerl et al., 2020) |
| Quinine | MDCK | MPP^+^ | 3.5±7.6 |  | (Hubeny et al., 2016) |
| Quinine | CHO and HEK293 | Ethidium | 70±10 |  | (Lee et al., 2009) |
| Quinine | HEK293 | MPP^+^ | 13±0.8 |  | (Müller et al., 2005) |
| Quinine | HEK293 | ASP^+^ | 96.2 |  | (Tzvetkov et al., 2012) |
| Quinine | HEK293 | ASP^+^ |  | * | (van der Velden et al., 2017) |
| Quinine | HeLa | TEA | 22.9 |  | (Zhang et al., 1998) |
| Rabeprazol | HEK293 | Metformin | 3.0±1.7 |  | (Nies et al., 2011) |
| Ranitidine | HEK293 | Trospium chloride | 186±25 |  | (Abebe et al., 2020) |
| Ranitidine | HeLa | TEA | 21.7±2.33 |  | (Bednarczyk et al., 2003) |
| Ranitidine | *Xenopus* oocytes | MPP^+^ | 33±5 |  | (Bourdet et al., 2005) |
| Ranitidine | HEK293 | MPP^+^ |  | * | (Boxberger et al., 2018) |
| Ranitidine | HEK293 | Serotonin |  | * | (Boxberger et al., 2018) |
| Ranitidine | HEK293 | MPP^+^ | 28±2 |  | (Müller et al., 2005) |
| Ranitidine | HEK293 | MPP^+^ | 42.0 |  | (Umehara et al., 2007) |
| Ranitidine | HEK293 | TEA | 23.0 |  | (Umehara et al., 2007) |
| Repaglinide | HEK293 | ASP^+^ | 9.2 |  | (Ahlin et al., 2008) |
| Repaglinide | MDCK | Metformin | 1.6 |  | (Bachmakov et al., 2008) |
| Repaglinide | MDCK | MPP^+^ | 1.8 |  | (Bachmakov et al., 2008) |
| Retrorsine | MDCK | MPP^+^ | 2.25±0.3 |  | (Tu et al., 2014) |
| Rhodamine 123 | HEK293 | TEA | 0.37±0.07 |  | (Jouan et al., 2014) |
| Rifabutin | HEK293 | Metformin | 38.7±3.8 |  | (Parvez et al., 2016) |
| Rifabutin | HEK293 | MPP^+^ | 42.7±6.4 |  | (Parvez et al., 2016) |
| Rifampicin | HEK293 | MPP^+^ | 603 |  | (Bi et al., 2019) |
| Rifamycin SV | HEK293 | MPP^+^ | 369 |  | (Bi et al., 2019) |
| Rilpivirine | KCL22 | TEA |  | * | (Moss et al., 2015) |
| Ritonavir | HEK293 | ASP^+^ | 11.2±3.5 |  | (Duan et al., 2015) |
| Ritonavir | HEK293 | Serotonin |  | * | (Duan et al., 2015) |
| Ritonavir | HEK293 | MPP^+^ | 14±2 |  | (Jung et al., 2008) |
| Ritonavir | KCL22 | TEA |  | * | (Moss et al., 2015) |
| Ritonavir | HEK293 | Metformin | 2.2±0.3 |  | (Panfen et al., 2019) |
| Ritonavir | HEK293 | Metformin | 1.1±0.1 |  | (Panfen et al., 2019) |
| Ritonavir | HEK293 | TEA | 4.1±0.6 |  | (Vermeer et al., 2016) |
| Ritonavir | HEK293 | ASP^+^ | 33.9 |  | (Wittwer et al., 2013) |
| Ritonavir | HeLa | TEA | 5.18±1.21 |  | (Zhang et al., 2000) |
| Rizatriptan | HEK293 | MPP^+^ | 41.0 |  | (Matthaei et al., 2016) |
| Ro 31-7549 | HEK293 | TEA |  | * | (Mayati et al., 2015) |
| Ro 31-8220 | HEK293 | TEA |  | * | (Mayati et al., 2015) |
| Ro31-8220 | HEK293 | DAPI |  | * | (Mayati et al., 2015) |
| Ro31-8220 | HEK293 | TEA | 0.18±0.05 |  | (Mayati et al., 2015) |
| Rocuronium | *Xenopus* oocytes | Tributylmethylammonium |  | * | (van Montfoort et al., 2001) |
| Rosiglitazone | MDCK | Metformin | 6.9 |  | (Bachmakov et al., 2008) |
| Rosiglitazone | MDCK | MPP^+^ | 30.4 |  | (Bachmakov et al., 2008) |
| Ruboxistaurin | HEK293 | TEA |  | * | (Mayati et al., 2015) |
| Rucaparib | CHO | Metformin | 4.3 |  | (Liao et al., 2020) |
| Rufloxacin | HEK293 | TEA |  | * | (Mulgaonkar et al., 2013) |
| Rutin | MDCK | DAPI |  | * | (Yasujima et al., 2011) |
| Salbutamol | HEK293 | MPP^+^ |  | * | (Salomon et al., 2015) |
| Salmeterol | HEK293 | MPP^+^ | 47.8 |  | (Salomon et al., 2015) |
| Saquinavir | HEK293 | ASP^+^ | 44.8±12.0 |  | (Duan et al., 2015) |
| Saquinavir | HEK293 | Serotonin |  | * | (Duan et al., 2015) |
| Saquinavir | HEK293 | MPP^+^ | 37±7 |  | (Jung et al., 2008) |
| Saquinavir | HeLa | TEA | 8.26±1.64 |  | (Zhang et al., 2000) |
| Saracatinib | HEK293 | ASP^+^ | 57 |  | (Harrach et al., 2017) |
| Saracatinib | HEK | ASP^+^ | 27.1 |  | (Morrow et al., 2010) |
| Scopolamine | HEK293 | MPP^+^ | 6.7±0.2 |  | (Chen et al., 2017b) |
| Serotonin | MDCK | DAPI |  | * | (Yasujima et al., 2011) |
| SKF525A | HEK293 | MPP^+^ | 6 ± 1 |  | (Cheong et al., 2017) |
| SKF550 | HEK293 | MPP^+^ | >0.3 |  | (Hayer-Zillgen et al., 2002) |
| Solutol HS15 | MDCK | MPP^+^ | 0.008 % (w/v) |  | (Otter et al., 2017) |
| Sorafenib | HEK293 | Metformin | >30 |  | (Minematsu and Giacomini, 2011) |
| Sparfloxacin | HEK293 | TEA | 94±8 |  | (Mulgaonkar et al., 2013) |
| Sparteine | HEK293 | TEA | 6.5±1.0 |  | (Neul et al., 2021) |
| Spironolacton | HEK293 | ASP^+^ |  | * | (Ahlin et al., 2008) |
| Spironolactone | HEK293 | ASP^+^ | 3.08±1.12 |  | (Ahlin et al., 2011) |
| Spironolactone | MDCK | MPP^+^ | 1.2 |  | (Grube et al., 2011) |
| Stavudine | KCL22 | TEA |  | * | (Moss et al., 2015) |
| Sufentanil |  | ASP^+^ | 19.36±0.2 | PAMPA | (Meyer et al., 2019) |
| Sulindac | S2 | TEA |  | * | (Khamdang et al., 2002) |
| Sulpiride | HEK293 | TEA | 182±42 |  | (Takano et al., 2017) |
| Sumatriptan | HEK293 | MPP^+^ | 46.5 |  | (Matthaei et al., 2016) |
| Sunitinib | HEK293 | ASP^+^ | 6.10 |  | (Chen et al., 2017a) |
| Sunitinib | HEK293 | MPP^+^ | 67±7 |  | (Johnston et al., 2014) |
| Sunitinib | HEK293 | Metformin | 0.33±0.016 |  | (Minematsu and Giacomini, 2011) |
| Tabentadol |  | ASP^+^ | 21.89±1.2 | PAMPA | (Meyer et al., 2019) |
| Tacrine | HEK293 | ASP^+^ | 21.72 |  | (Chen et al., 2017a) |
| Tacrine | HEK293 | ASP^+^ | 83 |  | (Kido et al., 2011) |
| Talinolol | MDCK | MPP^+^ | 23.7 |  | (Grube et al., 2011) |
| Tamoxifen | HEK293 | ASP^+^ |  | * | (Ahlin et al., 2008) |
| Taurocholate | *Xenopus* oocytes |  |  | * | (Zhang et al., 1997) |
| Telaprevir | HEK293 | MPP^+^ | 20.67±7.74 |  | (Kunze et al., 2012) |
| Tenatoprazole | HEK293 | Metformin | 23.3±7.1 |  | (Nies et al., 2011) |
| Tenofovir | CHO | MPP^+^ | 0.854±0.012 nM |  | (Minuesa et al., 2009) |
| Tenofovir | CHO | MPP^+^ |  |  | (Shen et al., 2017) |
| Terazosine | HEK293 | ASP^+^ | 23.7 |  | (Ahlin et al., 2008) |
| Terfenadine | HEK293 | ASP^+^ |  | * | (Ahlin et al., 2008) |
| Tetrabrombisphenol A | HEK293 | TEA | 37.5±1.2 |  | (Bruyère et al., 2017) |
| Tetrabutylammonium | HeLa | TEA | 6.5±1.5 |  | (Bednarczyk et al., 2003) |
| Tetrabutylammonium | *Xenopus* oocytes | MPP^+^ | 29.6±3.6 |  | (Dresser et al., 2002) |
| Tetraethylammonium (TEA) | *Xenopus* oocytes | MPP^+^ | 216 |  | (Bourdet et al., 2005) |
| Tetraethylammonium (TEA) | CHO | ASP^+^ | 673 |  | (Ciarimboli et al., 2004) |
| Tetraethylammonium (TEA) | HEK293 | ASP^+^ | 111 |  | (Ciarimboli et al., 2004) |
| Tetraethylammonium (TEA) | *Xenopus* oocytes | MPP^+^ | 158±40.5 |  | (Dresser et al., 2002) |
| Tetraethylammonium (TEA) | HEK294 | Propamocarb |  | * | (Gueniche et al., 2020) |
| Tetraethylammonium (TEA) | HEK293 | Aminoguanidine | 1390±60 |  | (Kimura et al., 2009) |
| Tetraethylammonium (TEA) | CHO and HEK293 | Ethidium | 7410±2380 |  | (Lee et al., 2009) |
| Tetraethylammonium (TEA) | CHO | MPP^+^ | 469.7±12.7 |  | (Ming et al., 2009) |
| Tetraethylammonium (TEA) | HEK293 | Trimethylamine N-oxide |  | * | (Miyake et al., 2017) |
| Tetraethylammonium (TEA) | HEK293 | Sulpiride |  | * | (Takano et al., 2017) |
| Tetraethylammonium (TEA) | HEK293 | MPP^+^ | 355 |  | (Umehara et al., 2007) |
| Tetraethylammonium (TEA) | *Xenopus* oocytes | MPP^+^ | 173±8.76 |  | (Zhang et al., 1997) |
| Tetramethrin | HEK293 | Dopamine |  | * | (Chedik et al., 2017) |
| Tetramethrin | HEK293 | TEA | 4.9 |  | (Chedik et al., 2017) |
| Tetramethylammonium | *Xenopus* oocytes | MPP^+^ | 12400±1280 |  | (Dresser et al., 2002) |
| Tetramethylguanidine | HEK293 | TEA | 480±80 |  | (Kimura et al., 2009) |
| Tetrapentyl ammonium | HEK293 | Pazopanib |  | * | (Ellawatty et al., 2018) |
| Tetrapentyl ammonium | HEK293 | ASP^+^ | 6.0±0.8 |  | (Huber et al., 2015) |
| Tetrapentylammonium | HeLa | TEA | 1.8±0.2 |  | (Bednarczyk et al., 2003) |
| Tetrapentylammonium | CHO | ASP^+^ | 5.5 |  | (Ciarimboli et al., 2004) |
| Tetrapentylammonium | HEK293 | ASP^+^ | 7.9 |  | (Ciarimboli et al., 2004) |
| Tetrapentylammonium | HeLa | TEA | 7.46 |  | (Zhang et al., 1998) |
| Tetrapropylammonium | HeLa | TEA | 22.0±4.1 |  | (Bednarczyk et al., 2003) |
| Tetrapropylammonium | *Xenopus* oocytes | MPP^+^ | 102±13.0 |  | (Dresser et al., 2002) |
| Thiamine | HeLa | TEA | 434±31.5 |  | (Bednarczyk et al., 2003) |
| Thiamine | HEK293 | MPP^+^ |  | * | (Boxberger et al., 2018) |
| Thiamine | HEK293 | Serotonin |  | * | (Boxberger et al., 2018) |
| Thiamine | HEK293 | TEA |  | * | (Boxberger et al., 2018) |
| Thiamine | HEK293 | ASP^+^ | 4354 |  | (Chen et al., 2017a) |
| Tilidine |  | ASP^+^ | 38.71±14.0 | PAMPA | (Meyer et al., 2019) |
| Tipranavir | HEK293 | ASP^+^ | 38.0±5.8 |  | (Duan et al., 2015) |
| Tipranavir | HEK293 | Serotonin |  | * | (Duan et al., 2015) |
| Tramadol | HEK293 | ASP^+^ | 52.5 |  | (Ahlin et al., 2008) |
| Tramadol | HEK293 | ASP^+^ | 30.3±3.1 |  | (Tzvetkov et al., 2011) |
| Trihexyphenidyl | HEK293 | ASP^+^ |  | * | (Ahlin et al., 2008) |
| Trimethoprim | HEK293 | ASP^+^ | 56.8 |  | (Ahlin et al., 2008) |
| Trimethoprim | HEK293 | ASP^+^ | 50.68 |  | (Chen et al., 2017a) |
| Trimethoprim | HEK293 | Quercetin |  | * | (Glaeser et al., 2014) |
| Trimethoprim | HEK293 | MPP^+^ | 20±3 |  | (Jung et al., 2008) |
| Trimethoprim | HEK293 | Nadolol |  | * | (Misaka et al., 2016) |
| Trimethoprim | HEK293 | Metformin | 9.6±1.2 |  | (Panfen et al., 2019) |
| Trimethoprim | HEK293 | Metformin | 9.2±0.7 |  | (Panfen et al., 2019) |
| Trimethoprim | MDCK | DAPI |  | * | (Yasujima et al., 2011) |
| Trimethoprim | MDCK | TEA |  | * | (Yasujima et al., 2011) |
| Trimethoprim | HEK293 | Metformin | 36.7±2.0 |  | (Müller et al., 2015) |
| Trimipramine | HEK293 | ASP^+^ | 27.7 |  | (Ahlin et al., 2008) |
| Trofosfamide | CHO | MPP^+^ |  | * | (Gupta et al., 2012) |
| Tropisetron | HEK293 | ASP^+^ | 8,5±1,4 |  | (Tzvetkov et al., 2012) |
| Tropisetron | HEK293 | Morphine | 3.3±0.6 |  | (Tzvetkov et al., 2013) |
| Trospium | HEK293 | MPP^+^ | 5.3 |  | (Bexten et al., 2015) |
| Trospium | HEK293 | MPP^+^ | 15.4±0.5 |  | (Chen et al., 2017b) |
| Trospium | HEK293 | MPP^+^ | 18.1±0.07 |  | (Wenge et al., 2011) |
| Tubocurarine | HEK293 | YM155 | 62.4 |  | (Minematsu et al., 2010) |
| Tween 20 | MDCK | MPP^+^ | 0.002 % (w/v) |  | (Otter et al., 2017) |
| Tween 80 | MDCK | MPP^+^ | 0.0007 % (w/v) |  | (Otter et al., 2017) |
| Tyramine | HeLa | TEA | 107±6.7 |  | (Bednarczyk et al., 2003) |
| Tyramine | HEK293 | MPP^+^ | 85.2±25.7 |  | (Seitz et al., 2015) |
| Vandetanib | HEK293 | MPP^+^ | 68±1 |  | (Johnston et al., 2014) |
| Vecuronium | *Xenopus* oocytes | MPP^+^ | 127±41.2 |  | (Zhang et al., 1997) |
| Vecuronium | HeLa | TEA | 232±7.63 |  | (Zhang et al., 1998) |
| Verapamil | HEK293 | ASP^+^ | 12.5±1.1 |  | (Ahlin et al., 2011) |
| Verapamil | HEK293 | Trospium chloride | 0.9 |  | (Bexten et al., 2015) |
| Verapamil | HEK293 | Serotonin | 1.5±1.4 |  | (Boxberger et al., 2014) |
| Verapamil | MDCK | Dehydrocordaline |  | * | (Chen et al., 2020) |
| Verapamil | HEK293 | Metformin |  | * | (Floerl et al., 2020) |
| Verapamil | HEK293 | MPP^+^ |  | * | (Floerl et al., 2020) |
| Verapamil | MDCK | MPP^+^ | 1.2 |  | (Grube et al., 2011) |
| Verapamil | HEK293 | Rhodamine 123 | 33.2±4.6 |  | (Jouan et al., 2014) |
| Verapamil | MDCK | Berberrubine |  | * | (Li et al., 2016) |
| Verapamil | MDCK | Coptisine |  | * | (Li et al., 2016) |
| Verapamil | MDCK | Epiberberine |  | * | (Li et al., 2016) |
| Verapamil | MDCK | Jatrorrhizine |  | * | (Li et al., 2016) |
| Verapamil | HEK293 | YM155 | 1.23 |  | (Minematsu et al., 2010) |
| Verapamil | HEK293 | Morphine | 1.6±0.3 |  | (Tzvetkov et al., 2013) |
| Verapamil | HEK293 | Metformin | 0.8±0.2 |  | (Parvez et al., 2016) |
| Verapamil | HEK293 | MPP^+^ | 1.1±0.2 |  | (Parvez et al., 2016) |
| Verapamil | HEK293 | para-Aminosalicylic acid | 1.2±0.35 |  | (Parvez et al., 2017) |
| Verapamil | HEK293 | Ethambutol | 2.0±0.2 |  | (Parvez et al., 2018) |
| Verapamil | HEK293 | Prothionamide | 3.9±0.4 |  | (Parvez et al., 2018) |
| Verapamil | MDCK | DAPI |  | * | (Yasujima et al., 2011) |
| Verapamil | MDCK | TEA |  | * | (Yasujima et al., 2011) |
| Verapamil | HeLa | TEA | 2.9 |  | (Zhang et al., 1998) |
| Verapamil | HEK293 | Morphine | 9.62±3.63 |  | (Zhu et al., 2018) |
| Vinblastine | CHO | MPP^+^ |  | * | (Gupta et al., 2012) |
| YM155 | HEK293 | MPP^+^ | 23.4 |  | (Minematsu et al., 2010) |
| YM758 | HEK293 | MPP^+^ | 40.5 |  | (Umehara et al., 2008) |
| Zalcitabine | HEK293 | MPP^+^ | 24±5 |  | (Jung et al., 2008) |
| Zalcitabine | KCL22 | TEA |  | * | (Moss et al., 2015) |
| Zearalenone | S2 | TEA | 0.62 |  | (Tachampa et al., 2008) |
| Zolmitriptan | HEK293 | MPP^+^ | >1000 |  | (Matthaei et al., 2016) |
| α-Zearalenol | S2 | TEA | 1.7 |  | (Tachampa et al., 2008) |
| β-Estradiol | HEK293 | ASP^+^ |  | * | (Ahlin et al., 2008) |
| β-Estradiol | HEK293 | MPP^+^ | 5.73±3.64 |  | (Hayer-Zillgen et al., 2002) |
| If not stated otherwise, the IC_50_ values are in µM as indicated in the headings. The * indicates that the tested inhibitor significantly (p < 0.05) reduced the uptake of the substrate into the single-transfected cell line to ≤ 50 %. | | | | | |

Abebe, B.T., Weiss, M., Modess, C., Tadken, T., Wegner, D., Meyer, M.J., et al. (2020). Pharmacokinetic drug-drug interactions between trospium chloride and ranitidine substrates of organic cation transporters in healthy human subjects. *J Clin Pharmacol* 60**,** 312-323. 10.1002/jcph.1523

Ahlin, G., Chen, L., Lazorova, L., Chen, Y., Ianculescu, A.G., Davis, R.L., et al. (2011). Genotype-dependent effects of inhibitors of the organic cation transporter, OCT1: predictions of metformin interactions. *Pharmacogenomics J* 11**,** 400-411. 10.1038/tpj.2010.54

Ahlin, G., Karlsson, J., Pedersen, J.M., Gustavsson, L., Larsson, R., Matsson, P., et al. (2008). Structural requirements for drug inhibition of the liver specific human organic cation transport protein 1. *J Med Chem* 51**,** 5932-5942. 10.1021/jm8003152

Amphoux, A., Vialou, V., Drescher, E., Brüss, M., Mannoury La Cour, C., Rochat, C., et al. (2006). Differential pharmacological in vitro properties of organic cation transporters and regional distribution in rat brain. *Neuropharmacology* 50**,** 941-952. 10.1016/j.neuropharm.2006.01.005

Arimany-Nardi, C., Minuesa, G., Keller, T., Erkizia, I., Koepsell, H., Martinez-Picado, J., et al. (2016). Role of human organic cation transporter 1 (hOCT1) polymorphisms in lamivudine (3TC) uptake and drug-drug interactions. *Front Pharmacol* 7**,** 175. 10.3389/fphar.2016.00175

Bachmakov, I., Glaeser, H., Fromm, M.F., and König, J. (2008). Interaction of oral antidiabetic drugs with hepatic uptake transporters: focus on organic anion transporting polypeptides and organic cation transporter 1. *Diabetes* 57**,** 1463-1469. 10.2337/db07-1515

Bednarczyk, D., Ekins, S., Wikel, J.H., and Wright, S.H. (2003). Influence of molecular structure on substrate binding to the human organic cation transporter, hOCT1. *Mol Pharmacol* 63**,** 489-498. 10.1124/mol.63.3.489

Bexten, M., Oswald, S., Grube, M., Jia, J., Graf, T., Zimmermann, U., et al. (2015). Expression of drug transporters and drug metabolizing enzymes in the bladder urothelium in man and affinity of the bladder spasmolytic trospium chloride to transporters likely involved in its pharmacokinetics. *Mol Pharm* 12**,** 171-178. 10.1021/mp500532x

Bi, Y.A., Costales, C., Mathialagan, S., West, M., Eatemadpour, S., Lazzaro, S., et al. (2019). Quantitative contribution of six major transporters to the hepatic uptake of drugs: "SLC-Phenotyping" using primary human hepatocytes. *J Pharmacol Exp Ther* 370**,** 72-83. 10.1124/jpet.119.257600

Bourdet, D.L., Pritchard, J.B., and Thakker, D.R. (2005). Differential substrate and inhibitory activities of ranitidine and famotidine toward human organic cation transporter 1 (hOCT1; SLC22A1), hOCT2 (SLC22A2), and hOCT3 (SLC22A3). *J Pharmacol Exp Ther* 315**,** 1288-1297. 10.1124/jpet.105.091223

Boxberger, K.H., Hagenbuch, B., and Lampe, J.N. (2014). Common drugs inhibit human organic cation transporter 1 (OCT1)-mediated neurotransmitter uptake. *Drug Metab Dispos* 42**,** 990-995. 10.1124/dmd.113.055095

Boxberger, K.H., Hagenbuch, B., and Lampe, J.N. (2018). Ligand-dependent modulation of hOCT1 transport reveals discrete ligand binding sites within the substrate translocation channel. *Biochem Pharmacol* 156**,** 371-384. 10.1016/j.bcp.2018.08.028

Bruyère, A., Hubert, C., Le Vee, M., Chedik, L., Sayyed, K., Stieger, B., et al. (2017). Inhibition of SLC drug transporter activities by environmental bisphenols. *Toxicol In Vitro* 40**,** 34-44. 10.1016/j.tiv.2016.12.009

Ceckova, M., Reznicek, J., Deutsch, B., Fromm, M.F., and Staud, F. (2018). Efavirenz reduces renal excretion of lamivudine in rats by inhibiting organic cation transporters (OCT, Oct) and multidrug and toxin extrusion proteins (MATE, Mate). *PLoS One* 13**,** e0202706. 10.1371/journal.pone.0202706

Ceckova, M., Reznicek, J., Ptackova, Z., Cerveny, L., Müller, F., Kacerovsky, M., et al. (2016). Role of ABC and solute carrier transporters in the placental transport of lamivudine. *Antimicrob Agents Chemother* 60**,** 5563-5572. 10.1128/AAC.00648-16

Chedik, L., Bruyère, A., and Fardel, O. (2019). Interactions of organophosphorus pesticides with solute carrier (SLC) drug transporters. *Xenobiotica* 49**,** 363-374. 10.1080/00498254.2018.1442030

Chedik, L., Bruyere, A., Le Vee, M., Stieger, B., Denizot, C., Parmentier, Y., et al. (2017). Inhibition of human drug transporter activities by the pyrethroid pesticides allethrin and tetramethrin. *PLoS One* 12**,** e0169480. 10.1371/journal.pone.0169480

Chen, E.C., Khuri, N., Liang, X., Stecula, A., Chien, H.C., Yee, S.W., et al. (2017a). Discovery of competitive and noncompetitive ligands of the organic cation transporter 1 (OCT1; SLC22A1). *J Med Chem* 60**,** 2685-2696. 10.1021/acs.jmedchem.6b01317

Chen, J., Brockmöller, J., Seitz, T., König, J., Chen, X., and Tzvetkov, M.V. (2017b). Tropane alkaloids as substrates and inhibitors of human organic cation transporters of the SLC22 (OCT) and the SLC47 (MATE) families. *Biol Chem* 398**,** 237-249. 10.1515/hsz-2016-0236

Chen, J.Y., Brockmöller, J., Tzvetkov, M.V., Wang, L.J., and Chen, X.J. (2019). An *in vitro* study on interaction of anisodine and monocrotaline with organic cation transporters of the SLC22 and SLC47 families. *Chin J Nat Med* 17**,** 490-497. 10.1016/S1875-5364(19)30070-6

Chen, L., Shu, Y., Liang, X., Chen, E.C., Yee, S.W., Zur, A.A., et al. (2014). OCT1 is a high-capacity thiamine transporter that regulates hepatic steatosis and is a target of metformin. *Proc Natl Acad Sci U S A* 111**,** 9983-9988. 10.1073/pnas.1314939111

Chen, M., Neul, C., Schaeffeler, E., Frisch, F., Winter, S., Schwab, M., et al. (2020). Sorafenib activity and disposition in liver cancer does not depend on organic cation transporter 1. *Clin Pharmacol Ther* 107**,** 227-237. 10.1002/cpt.1588

Cheong, J., Halladay, J.S., Plise, E., Sodhi, J.K., and Salphati, L. (2017). The effects of drug metabolizing enzyme inhibitors on hepatic efflux and uptake transporters. *Drug Metab Lett* 11**,** 111-118. 10.2174/1872312811666171010101248

Chevalier, C., Fouqueray, P., and Bolze, S. (2020). In vitro investigation, pharmacokinetics and disposition of imeglimin, a novel oral antidiabetic drug, in preclinical species and humans. *Drug Metab Dispos*. 10.1124/dmd.120.000154

Ciarimboli, G., Struwe, K., Arndt, P., Gorboulev, V., Koepsell, H., Schlatter, E., et al. (2004). Regulation of the human organic cation transporter hOCT1. *J Cell Physiol* 201**,** 420-428. 10.1002/jcp.20081

Dickens, D., Owen, A., Alfirevic, A., Giannoudis, A., Davies, A., Weksler, B., et al. (2012). Lamotrigine is a substrate for OCT1 in brain endothelial cells. *Biochem Pharmacol* 83**,** 805-814. 10.1016/j.bcp.2011.12.032

Dresser, M.J., Xiao, G., Leabman, M.K., Gray, A.T., and Giacomini, K.M. (2002). Interactions of *n*-tetraalkylammonium compounds and biguanides with a human renal organic cation transporter (hOCT2). *Pharm Res* 19**,** 1244-1247. 10.1023/a:1019870831174

Duan, H., Hu, T., Foti, R.S., Pan, Y., Swaan, P.W., and Wang, J. (2015). Potent and selective inhibition of plasma membrane monoamine transporter by HIV protease inhibitors. *Drug Metab Dispos* 43**,** 1773-1780. 10.1124/dmd.115.064824

Ellawatty, W.E.A., Masuo, Y., Fujita, K.I., Yamazaki, E., Ishida, H., Arakawa, H., et al. (2018). Organic cation transporter 1 is responsible for hepatocellular uptake of the tyrosine kinase inhibitor pazopanib. *Drug Metab Dispos* 46**,** 33-40. 10.1124/dmd.117.076554

Floerl, S., Kuehne, A., and Hagos, Y. (2020). Functional and pharmacological comparison of human, mouse, and rat organic cation transporter 1 toward drug and pesticide interaction. *Int J Mol Sci* 21. 10.3390/ijms21186871

Glaeser, H., Bujok, K., Schmidt, I., Fromm, M.F., and Mandery, K. (2014). Organic anion transporting polypeptides and organic cation transporter 1 contribute to the cellular uptake of the flavonoid quercetin. *Naunyn Schmiedebergs Arch Pharmacol* 387**,** 883-891. 10.1007/s00210-014-1000-6

Grube, M., Ameling, S., Noutsias, M., Köck, K., Triebel, I., Bonitz, K., et al. (2011). Selective regulation of cardiac organic cation transporter novel type 2 (OCTN2) in dilated cardiomyopathy. *Am J Pathol* 178**,** 2547-2559. 10.1016/j.ajpath.2011.02.020

Gründemann, D., Hahne, C., Berkels, R., and Schömig, E. (2003). Agmatine is efficiently transported by non-neuronal monoamine transporters extraneuronal monoamine transporter (EMT) and organic cation transporter 2 (OCT2). *J Pharmacol Exp Ther* 304**,** 810-817. 10.1124/jpet.102.044404

Gueniche, N., Bruyere, A., Ringeval, M., Jouan, E., Huguet, A., Le Hégarat, L., et al. (2020). Differential interactions of carbamate pesticides with drug transporters. *Xenobiotica* 50**,** 1380-1392. 10.1080/00498254.2020.1771473

Gupta, S., Wulf, G., Henjakovic, M., Koepsell, H., Burckhardt, G., and Hagos, Y. (2012). Human organic cation transporter 1 is expressed in lymphoma cells and increases susceptibility to irinotecan and paclitaxel. *J Pharmacol Exp Ther* 341**,** 16-23. 10.1124/jpet.111.190561

Harrach, S., Edemir, B., Schmidt-Lauber, C., Pap, T., Bertrand, J., and Ciarimboli, G. (2017). Importance of the novel organic cation transporter 1 for tyrosine kinase inhibition by saracatinib in rheumatoid arthritis synovial fibroblasts. *Sci Rep* 7**,** 1258. 10.1038/s41598-017-01438-4

Hayer-Zillgen, M., Brüss, M., and Bönisch, H. (2002). Expression and pharmacological profile of the human organic cation transporters hOCT1, hOCT2 and hOCT3. *Br J Pharmacol* 136**,** 829-836. 10.1038/sj.bjp.0704785

Hubeny, A., Keiser, M., Oswald, S., Jedlitschky, G., Kroemer, H.K., Siegmund, W., et al. (2016). Expression of organic anion transporting polypeptide 1A2 in red blood cells and its potential impact on antimalarial therapy. *Drug Metab Dispos* 44**,** 1562-1568. 10.1124/dmd.116.069807

Huber, S., Huettner, J.P., Hacker, K., Bernhardt, G., Konig, J., and Buschauer, A. (2015). Esters of Bendamustine Are by Far More Potent Cytotoxic Agents than the Parent Compound against Human Sarcoma and Carcinoma Cells. *PLoS One* 10**,** e0133743. 10.1371/journal.pone.0133743

Ishiguro, N., Shimizu, H., Kishimoto, W., Ebner, T., and Schaefer, O. (2013). Evaluation and prediction of potential drug-drug interactions of linagliptin using in vitro cell culture methods. *Drug Metab Dispos* 41**,** 149-158. 10.1124/dmd.112.048470

Ito, S., Kusuhara, H., Kuroiwa, Y., Wu, C., Moriyama, Y., Inoue, K., et al. (2010). Potent and specific inhibition of mMate1-mediated efflux of type I organic cations in the liver and kidney by pyrimethamine. *J Pharmacol Exp Ther* 333**,** 341-350. 10.1124/jpet.109.163642

Johnston, R.A., Rawling, T., Chan, T., Zhou, F., and Murray, M. (2014). Selective inhibition of human solute carrier transporters by multikinase inhibitors. *Drug Metab Dispos* 42**,** 1851-1857. 10.1124/dmd.114.059097

Jouan, E., Le Vee, M., Denizot, C., Da Violante, G., and Fardel, O. (2014). The mitochondrial fluorescent dye rhodamine 123 is a high-affinity substrate for organic cation transporters (OCTs) 1 and 2. *Fundam Clin Pharmacol* 28**,** 65-77. 10.1111/j.1472-8206.2012.01071.x

Jung, N., Lehmann, C., Rubbert, A., Knispel, M., Hartmann, P., Van Lunzen, J., et al. (2008). Relevance of the organic cation transporters 1 and 2 for antiretroviral drug therapy in human immunodeficiency virus infection. *Drug Metab Dispos* 36**,** 1616-1623. 10.1124/dmd.108.020826

Khamdang, S., Takeda, M., Noshiro, R., Narikawa, S., Enomoto, A., Anzai, N., et al. (2002). Interactions of human organic anion transporters and human organic cation transporters with nonsteroidal anti-inflammatory drugs. *J Pharmacol Exp Ther* 303**,** 534-539. 10.1124/jpet.102.037580

Kido, Y., Matsson, P., and Giacomini, K.M. (2011). Profiling of a prescription drug library for potential renal drug-drug interactions mediated by the organic cation transporter 2. *J Med Chem* 54**,** 4548-4558. 10.1021/jm2001629

Kimura, H., Takeda, M., Narikawa, S., Enomoto, A., Ichida, K., and Endou, H. (2002). Human organic anion transporters and human organic cation transporters mediate renal transport of prostaglandins. *J Pharmacol Exp Ther* 301**,** 293-298. 10.1124/jpet.301.1.293

Kimura, N., Masuda, S., Katsura, T., and Inui, K. (2009). Transport of guanidine compounds by human organic cation transporters, hOCT1 and hOCT2. *Biochem Pharmacol* 77**,** 1429-1436. 10.1016/j.bcp.2009.01.010

Knop, J., Misaka, S., Singer, K., Hoier, E., Müller, F., Glaeser, H., et al. (2015). Inhibitory effects of green tea and (-)-epigallocatechin gallate on transport by OATP1B1, OATP1B3, OCT1, OCT2, MATE1, MATE2-K and P-Glycoprotein. *PLoS One* 10**,** e0139370. 10.1371/journal.pone.0139370

Kunze, A., Huwyler, J., Camenisch, G., and Gutmann, H. (2012). Interaction of the antiviral drug telaprevir with renal and hepatic drug transporters. *Biochem Pharmacol* 84**,** 1096-1102. 10.1016/j.bcp.2012.07.032

Kwon, M., Choi, Y.A., Choi, M.K., and Song, I.S. (2015). Organic cation transporter-mediated drug-drug interaction potential between berberine and metformin. *Arch Pharm Res* 38**,** 849-856. 10.1007/s12272-014-0510-6

Lee, W.K., Reichold, M., Edemir, B., Ciarimboli, G., Warth, R., Koepsell, H., et al. (2009). Organic cation transporters OCT1, 2, and 3 mediate high-affinity transport of the mutagenic vital dye ethidium in the kidney proximal tubule. *Am J Physiol Renal Physiol* 296**,** F1504-1513. 10.1152/ajprenal.90754.2008

Li, L., Lei, H., Wang, W., Du, W., Yuan, J., Tu, M., et al. (2018). Co-administration of nuciferine reduces the concentration of metformin in liver via differential inhibition of hepatic drug transporter OCT1 and MATE1. *Biopharm Drug Dispos* 39**,** 411-419. 10.1002/bdd.2158

Li, L., Song, F., Tu, M., Wang, K., Zhao, L., Wu, X., et al. (2014a). *In vitro* interaction of clopidogrel and its hydrolysate with OCT1, OCT2 and OAT1. *Int J Pharm* 465**,** 5-10. 10.1016/j.ijpharm.2014.02.003

Li, L., Sun, S., Weng, Y., Song, F., Zhou, S., Bai, M., et al. (2016). Interaction of six protoberberine alkaloids with human organic cation transporters 1, 2 and 3. *Xenobiotica* 46**,** 175-183. 10.3109/00498254.2015.1056283

Li, L., Tu, M., Yang, X., Sun, S., Wu, X., Zhou, H., et al. (2014b). The contribution of human OCT1, OCT3, and CYP3A4 to nitidine chloride-induced hepatocellular toxicity. *Drug Metab Dispos* 42**,** 1227-1234. 10.1124/dmd.113.056689

Liang, R.F., Ge, W.J., Song, X.M., Zhang, J.P., Cui, W.F., Zhang, S.F., et al. (2020). Involvement of organic anion-transporting polypeptides and organic cation transporter in the hepatic uptake of jatrorrhizine. *Xenobiotica* 50**,** 479-487. 10.1080/00498254.2019.1651921

Liao, M., Jaw-Tsai, S., Beltman, J., Simmons, A.D., Harding, T.C., and Xiao, J.J. (2020). Evaluation of *in vitro* absorption, distribution, metabolism, and excretion and assessment of drug-drug interaction of rucaparib, an orally potent poly(ADP-ribose) polymerase inhibitor. *Xenobiotica* 50**,** 1032-1042. 10.1080/00498254.2020.1737759

Lips, K.S., Volk, C., Schmitt, B.M., Pfeil, U., Arndt, P., Miska, D., et al. (2005). Polyspecific cation transporters mediate luminal release of acetylcholine from bronchial epithelium. *Am J Respir Cell Mol Biol* 33**,** 79-88. 10.1165/rcmb.2004-0363OC

Lopez Quinones, A.J., Wagner, D.J., and Wang, J. (2020). Characterization of *meta*-iodobenzylguanidine (mIBG) transport by polyspecific organic cation transporters: implication for mIBG therapy. *Mol Pharmacol* 98**,** 109-119. 10.1124/mol.120.119495

Lucero, M.L., Gonzalo, A., Ganza, A., Leal, N., Soengas, I., Ioja, E., et al. (2012). Interactions of bilastine, a new oral H_1_ antihistamine, with human transporter systems. *Drug Chem Toxicol* 35 Suppl 1**,** 8-17. 10.3109/01480545.2012.682653

Luo, L., Ramanathan, R., Horlbogen, L., Mathialagan, S., Costales, C., Vourvahis, M., et al. (2020). A multiplexed HILIC-MS/HRMS assay for the assessment of transporter inhibition biomarkers in phase I clinical trials: isobutyryl-carnitine as an organic cation transporter (OCT1) biomarker. *Anal Chem* 92**,** 9745-9754. 10.1021/acs.analchem.0c01144

Mamidi, R., Dallas, S., Sensenhauser, C., Lim, H.K., Scheers, E., Verboven, P., et al. (2017). *In vitro* and physiologically-based pharmacokinetic based assessment of drug-drug interaction potential of canagliflozin. *Br J Clin Pharmacol* 83**,** 1082-1096. 10.1111/bcp.13186

Matthaei, J., Kuron, D., Faltraco, F., Knoch, T., Dos Santos Pereira, J.N., Abu Abed, M., et al. (2016). OCT1 mediates hepatic uptake of sumatriptan and loss-of-function OCT1 polymorphisms affect sumatriptan pharmacokinetics. *Clin Pharmacol Ther* 99**,** 633-641. 10.1002/cpt.317

Mayati, A., Bruyere, A., Moreau, A., Jouan, E., Denizot, C., Parmentier, Y., et al. (2015). Protein kinase C-independent inhibition of organic cation transporter 1 activity by the bisindolylmaleimide Ro 31-8220. *PLoS One* 10**,** e0144667. 10.1371/journal.pone.0144667

Mccormick, A., and Swaisland, H. (2017). *In vitro* assessment of the roles of drug transporters in the disposition and drug-drug interaction potential of olaparib. *Xenobiotica* 47**,** 903-915. 10.1080/00498254.2016.1241449

Meyer, M.J., Neumann, V.E., Friesacher, H.R., Zdrazil, B., Brockmöller, J., and Tzvetkov, M.V. (2019). Opioids as substrates and inhibitors of the genetically highly variable organic cation transporter OCT1. *J Med Chem* 62**,** 9890-9905. 10.1021/acs.jmedchem.9b01301

Milunović, M.N.M., Palamarciuc, O., Sirbu, A., Shova, S., Dumitrescu, D., Dvoranová, D., et al. (2020). Insight into the anticancer activity of copper(II) 5-methylenetrimethylammonium-thiosemicarbazonates and their interaction with organic cation transporters. *Biomolecules* 10. 10.3390/biom10091213

Mimura, Y., Yasujima, T., Ohta, K., Inoue, K., and Yuasa, H. (2015). Functional identification of organic cation transporter 1 as an atenolol transporter sensitive to flavonoids. *Biochem Biophys Rep* 2**,** 166-171. 10.1016/j.bbrep.2015.06.005

Minematsu, T., and Giacomini, K.M. (2011). Interactions of tyrosine kinase inhibitors with organic cation transporters and multidrug and toxic compound extrusion proteins. *Mol Cancer Ther* 10**,** 531-539. 10.1158/1535-7163.MCT-10-0731

Minematsu, T., Iwai, M., Umehara, K., Usui, T., and Kamimura, H. (2010). Characterization of human organic cation transporter 1 (OCT1/SLC22A1)- and OCT2 (SLC22A2)-mediated transport of 1-(2-methoxyethyl)-2-methyl-4,9-dioxo-3-(pyrazin-2-ylmethyl)- 4,9-dihydro-1*H*-naphtho[2,3-*d*]imidazolium bromide (YM155 monobromide), a novel small molecule survivin suppressant. *Drug Metab Dispos* 38**,** 1-4. 10.1124/dmd.109.028142

Ming, X., Ju, W., Wu, H., Tidwell, R.R., Hall, J.E., and Thakker, D.R. (2009). Transport of dicationic drugs pentamidine and furamidine by human organic cation transporters. *Drug Metab Dispos* 37**,** 424-430. 10.1124/dmd.108.024083

Minuesa, G., Volk, C., Molina-Arcas, M., Gorboulev, V., Erkizia, I., Arndt, P., et al. (2009). Transport of lamivudine [(-)-*β*-_L_-2',3'-dideoxy-3'-thiacytidine] and high-affinity interaction of nucleoside reverse transcriptase inhibitors with human organic cation transporters 1, 2, and 3. *J Pharmacol Exp Ther* 329**,** 252-261. 10.1124/jpet.108.146225

Misaka, S., Knop, J., Singer, K., Hoier, E., Keiser, M., Müller, F., et al. (2016). The nonmetabolized *β*-blocker nadolol is a substrate of OCT1, OCT2, MATE1, MATE2-K, and P-Glycoprotein, but not of OATP1B1 and OATP1B3. *Mol Pharm* 13**,** 512-519. 10.1021/acs.molpharmaceut.5b00733

Miyake, T., Mizuno, T., Mochizuki, T., Kimura, M., Matsuki, S., Irie, S., et al. (2017). Involvement of organic cation transporters in the kinetics of trimethylamine N-oxide. *J Pharm Sci* 106**,** 2542-2550. 10.1016/j.xphs.2017.04.067

Morrow, C.J., Ghattas, M., Smith, C., Bonisch, H., Bryce, R.A., Hickinson, D.M., et al. (2010). Src family kinase inhibitor saracatinib (AZD0530) impairs oxaliplatin uptake in colorectal cancer cells and blocks organic cation transporters. *Cancer Res* 70**,** 5931-5941. 10.1158/0008-5472.CAN-10-0694

Morse, B.L., Kolur, A., Hudson, L.R., Hogan, A.T., Chen, L.H., Brackman, R.M., et al. (2020). Pharmacokinetics of organic cation transporter 1 (OCT1) substrates in Oct1/2 knockout mice and species difference in hepatic OCT1-mediated uptake. *Drug Metab Dispos* 48**,** 93-105. 10.1124/dmd.119.088781

Moss, D.M., Liptrott, N.J., Siccardi, M., and Owen, A. (2015). Interactions of antiretroviral drugs with the SLC22A1 (OCT1) drug transporter. *Front Pharmacol* 6**,** 78. 10.3389/fphar.2015.00078

Mulgaonkar, A., Venitz, J., Gründemann, D., and Sweet, D.H. (2013). Human organic cation transporters 1 (SLC22A1), 2 (SLC22A2), and 3 (SLC22A3) as disposition pathways for fluoroquinolone antimicrobials. *Antimicrob Agents Chemother* 57**,** 2705-2711. 10.1128/AAC.02289-12

Müller, F., Pontones, C.A., Renner, B., Mieth, M., Hoier, E., Auge, D., et al. (2015). N^1^-methylnicotinamide as an endogenous probe for drug interactions by renal cation transporters: studies on the metformin-trimethoprim interaction. *Eur J Clin Pharmacol* 71**,** 85-94. 10.1007/s00228-014-1770-2

Müller, J., Lips, K.S., Metzner, L., Neubert, R.H., Koepsell, H., and Brandsch, M. (2005). Drug specificity and intestinal membrane localization of human organic cation transporters (OCT). *Biochem Pharmacol* 70**,** 1851-1860. 10.1016/j.bcp.2005.09.011

Neul, C., Hofmann, U., Schaeffeler, E., Winter, S., Klein, K., Giacomini, K.M., et al. (2021). Characterization of cytochrome P450 (CYP) 2D6 drugs as substrates of human organic cation transporters and multidrug and toxin extrusion proteins. *Br J Pharmacol*. 10.1111/bph.15370

Nies, A.T., Herrmann, E., Brom, M., and Keppler, D. (2008). Vectorial transport of the plant alkaloid berberine by double-transfected cells expressing the human organic cation transporter 1 (OCT1, SLC22A1) and the efflux pump MDR1 P-glycoprotein (ABCB1). *Naunyn Schmiedebergs Arch Pharmacol* 376**,** 449-461. 10.1007/s00210-007-0219-x

Nies, A.T., Hofmann, U., Resch, C., Schaeffeler, E., Rius, M., and Schwab, M. (2011). Proton pump inhibitors inhibit metformin uptake by organic cation transporters (OCTs). *PLoS One* 6**,** e22163. 10.1371/journal.pone.0022163

Nies, A.T., Koepsell, H., Winter, S., Burk, O., Klein, K., Kerb, R., et al. (2009). Expression of organic cation transporters OCT1 (SLC22A1) and OCT3 (SLC22A3) is affected by genetic factors and cholestasis in human liver. *Hepatology* 50**,** 1227-1240. 10.1002/hep.23103

Nies, A.T., Schaeffeler, E., Van Der Kuip, H., Cascorbi, I., Bruhn, O., Kneba, M., et al. (2014). Cellular uptake of imatinib into leukemic cells is independent of human organic cation transporter 1 (OCT1). *Clin Cancer Res* 20**,** 985-994. 10.1158/1078-0432.CCR-13-1999

Obianom, O.N., Coutinho, A.L., Yang, W., Yang, H., Xue, F., and Shu, Y. (2017). Incorporation of a biguanide scaffold enhances drug uptake by organic cation transporters 1 and 2. *Mol Pharm* 14**,** 2726-2739. 10.1021/acs.molpharmaceut.7b00285

Otter, M., Oswald, S., Siegmund, W., and Keiser, M. (2017). Effects of frequently used pharmaceutical excipients on the organic cation transporters 1-3 and peptide transporters 1/2 stably expressed in MDCKII cells. *Eur J Pharm Biopharm* 112**,** 187-195. 10.1016/j.ejpb.2016.11.028

Panfen, E., Chen, W., Zhang, Y., Sinz, M., Marathe, P., Gan, J., et al. (2019). Enhanced and persistent inhibition of organic cation transporter 1 activity by preincubation of cyclosporine A. *Drug Metab Dispos* 47**,** 1352-1360. 10.1124/dmd.119.087197

Parvez, M.M., Kaisar, N., Shin, H.J., Jung, J.A., and Shin, J.G. (2016). Inhibitory interaction potential of 22 antituberculosis drugs on organic anion and cation transporters of the SLC22A family. *Antimicrob Agents Chemother* 60**,** 6558-6567. 10.1128/AAC.01151-16

Parvez, M.M., Kaisar, N., Shin, H.J., Lee, Y.J., and Shin, J.G. (2018). Comprehensive substrate characterization of 22 antituberculosis drugs for multiple solute carrier (SLC) uptake transporters *in vitro*. *Antimicrob Agents Chemother* 62. 10.1128/AAC.00512-18

Parvez, M.M., Shin, H.J., Jung, J.A., and Shin, J.G. (2017). Evaluation of *para*-aminosalicylic acid as a substrate of multiple solute carrier uptake transporters and possible drug interactions with nonsteroidal anti-inflammatory drugs *in vitro*. *Antimicrob Agents Chemother* 61. 10.1128/AAC.02392-16

Reese, M.J., Bowers, G.D., Humphreys, J.E., Gould, E.P., Ford, S.L., Webster, L.O., et al. (2016). Drug interaction profile of the HIV integrase inhibitor cabotegravir: assessment from *in vitro* studies and a clinical investigation with midazolam. *Xenobiotica* 46**,** 445-456. 10.3109/00498254.2015.1081993

Saadatmand, A.R., Tadjerpisheh, S., Brockmöller, J., and Tzvetkov, M.V. (2012). The prototypic pharmacogenetic drug debrisoquine is a substrate of the genetically polymorphic organic cation transporter OCT1. *Biochem Pharmacol* 83**,** 1427-1434. 10.1016/j.bcp.2012.01.032

Salomon, J.J., Hagos, Y., Petzke, S., Kühne, A., Gausterer, J.C., Hosoya, K., et al. (2015). Beta-2 adrenergic agonists are substrates and inhibitors of human organic cation transporter 1. *Mol Pharm* 12**,** 2633-2641. 10.1021/mp500854e

Sayyed, K., Camillerapp, C., Le Vée, M., Bruyère, A., Nies, A.T., Abdel-Razzak, Z., et al. (2019). Inhibition of organic cation transporter (OCT) activities by carcinogenic heterocyclic aromatic amines. *Toxicol In Vitro* 54**,** 10-22. 10.1016/j.tiv.2018.08.015

Sayyed, K., Vee, M.L., Abdel-Razzak, Z., Jouan, E., Stieger, B., Denizot, C., et al. (2016). Alteration of human hepatic drug transporter activity and expression by cigarette smoke condensate. *Toxicology* 363-364**,** 58-71. 10.1016/j.tox.2016.07.011

Seitz, T., Stalmann, R., Dalila, N., Chen, J., Pojar, S., Dos Santos Pereira, J.N., et al. (2015). Global genetic analyses reveal strong inter-ethnic variability in the loss of activity of the organic cation transporter OCT1. *Genome Med* 7**,** 56. 10.1186/s13073-015-0172-0

Shams, T., Lu, X., Zhu, L., and Zhou, F. (2018). The inhibitory effects of five alkaloids on the substrate transport mediated through human organic anion and cation transporters. *Xenobiotica* 48**,** 197-205. 10.1080/00498254.2017.1282647

Shen, H., Li, W., Humphreys, W.G., and Lai, Y. (2017). Tenofovir disoproxil fumarate is not an inhibitor of human organic cation transporter 1. *J Pharmacol Exp Ther* 360**,** 341-342. 10.1124/jpet.116.238337

Shen, Z., Yeh, L.T., Wallach, K., Zhu, N., Kerr, B., and Gillen, M. (2016). In vitro and in vivo interaction studies between lesinurad, a selective urate reabsorption inhibitor, and major liver or kidney transporters. *Clin Drug Investig* 36**,** 443-452. 10.1007/s40261-016-0386-y

Shibata, M., Toyoshima, J., Kaneko, Y., Oda, K., and Nishimura, T. (2020). A drug-drug interaction study to evaluate the impact of peficitinib on OCT1- and MATE1-mediated transport of metformin in healthy volunteers. *Eur J Clin Pharmacol* 76**,** 1135-1141. 10.1007/s00228-020-02876-2

Tachampa, K., Takeda, M., Khamdang, S., Noshiro-Kofuji, R., Tsuda, M., Jariyawat, S., et al. (2008). Interactions of organic anion transporters and organic cation transporters with mycotoxins. *J Pharmacol Sci* 106**,** 435-443. 10.1254/jphs.fp0070911

Takano, H., Ito, S., Zhang, X., Ito, H., Zhang, M.R., Suzuki, H., et al. (2017). Possible role of organic cation transporters in the distribution of [^11^C]sulpiride, a dopamine D_2_ receptor antagonist. *J Pharm Sci* 106**,** 2558-2565. 10.1016/j.xphs.2017.05.006

Takeda, M., Khamdang, S., Narikawa, S., Kimura, H., Kobayashi, Y., Yamamoto, T., et al. (2002). Human organic anion transporters and human organic cation transporters mediate renal antiviral transport. *J Pharmacol Exp Ther* 300**,** 918-924. 10.1124/jpet.300.3.918

Te Brake, L.H., Van Den Heuvel, J.J., Buaben, A.O., Van Crevel, R., Bilos, A., Russel, F.G., et al. (2016). Moxifloxacin is a potent *in vitro* inhibitor of OCT- and MATE-mediated transport of metformin and ethambutol. *Antimicrob Agents Chemother* 60**,** 7105-7114. 10.1128/AAC.01471-16

Tu, M., Li, L., Lei, H., Ma, Z., Chen, Z., Sun, S., et al. (2014). Involvement of organic cation transporter 1 and CYP3A4 in retrorsine-induced toxicity. *Toxicology* 322**,** 34-42. 10.1016/j.tox.2014.04.007

Tu, M., Sun, S., Wang, K., Peng, X., Wang, R., Li, L., et al. (2013). Organic cation transporter 1 mediates the uptake of monocrotaline and plays an important role in its hepatotoxicity. *Toxicology* 311**,** 225-230. 10.1016/j.tox.2013.06.009

Tzvetkov, M.V., Dos Santos Pereira, J.N., Meineke, I., Saadatmand, A.R., Stingl, J.C., and Brockmöller, J. (2013). Morphine is a substrate of the organic cation transporter OCT1 and polymorphisms in *OCT1* gene affect morphine pharmacokinetics after codeine administration. *Biochem Pharmacol* 86**,** 666-678. 10.1016/j.bcp.2013.06.019

Tzvetkov, M.V., Matthaei, J., Pojar, S., Faltraco, F., Vogler, S., Prukop, T., et al. (2018). Increased systemic exposure and stronger cardiovascular and metabolic adverse reactions to fenoterol in individuals with heritable *OCT1* deficiency. *Clin Pharmacol Ther* 103**,** 868-878. 10.1002/cpt.812

Tzvetkov, M.V., Saadatmand, A.R., Bokelmann, K., Meineke, I., Kaiser, R., and Brockmöller, J. (2012). Effects of *OCT1* polymorphisms on the cellular uptake, plasma concentrations and efficacy of the 5-HT_3_ antagonists tropisetron and ondansetron. *Pharmacogenomics J* 12**,** 22-29. 10.1038/tpj.2010.75

Tzvetkov, M.V., Saadatmand, A.R., Lötsch, J., Tegeder, I., Stingl, J.C., and Brockmöller, J. (2011). Genetically polymorphic OCT1: another piece in the puzzle of the variable pharmacokinetics and pharmacodynamics of the opioidergic drug tramadol. *Clin Pharmacol Ther* 90**,** 143-150. 10.1038/clpt.2011.56

Umehara, K.I., Iwatsubo, T., Noguchi, K., and Kamimura, H. (2007). Functional involvement of organic cation transporter 1 (OCT1/Oct1) in the hepatic uptake of organic cations in humans and rats. *Xenobiotica* 37**,** 818-831. 10.1080/00498250701546012

Umehara, K.I., Iwatsubo, T., Noguchi, K., Usui, T., and Kamimura, H. (2008). Effect of cationic drugs on the transporting activity of human and rat OCT/Oct 1-3 *in vitro* and implications for drug-drug interactions. *Xenobiotica* 38**,** 1203-1218. 10.1080/00498250802334409

Van Der Velden, M., Bilos, A., Van Den Heuvel, J., Rijpma, S.R., Hurkmans, E.G.E., Sauerwein, R.W., et al. (2017). Proguanil and cycloguanil are organic cation transporter and multidrug and toxin extrusion substrates. *Malar J* 16**,** 422. 10.1186/s12936-017-2062-y

Van Montfoort, J.E., Müller, M., Groothuis, G.M., Meijer, D.K., Koepsell, H., and Meier, P.J. (2001). Comparison of "type I" and "type II" organic cation transport by organic cation transporters and organic anion-transporting polypeptides. *J Pharmacol Exp Ther* 298**,** 110-115.

Vermeer, L.M., Isringhausen, C.D., Ogilvie, B.W., and Buckley, D.B. (2016). Evaluation of ketoconazole and its alternative clinical CYP3A4/5 inhibitors as inhibitors of drug transporters: the in vitro effects of ketoconazole, ritonavir, clarithromycin, and itraconazole on 13 clinically-relevant drug transporters. *Drug Metab Dispos* 44**,** 453-459. 10.1124/dmd.115.067744

Wagner, D.J., Sager, J.E., Duan, H., Isoherranen, N., and Wang, J. (2017). Interaction and transport of methamphetamine and its primary metabolites by organic cation and multidrug and toxin extrusion transporters. *Drug Metab Dispos* 45**,** 770-778. 10.1124/dmd.116.074708

Wenge, B., Geyer, J., and Bönisch, H. (2011). Oxybutynin and trospium are substrates of the human organic cation transporters. *Naunyn Schmiedebergs Arch Pharmacol* 383**,** 203-208. 10.1007/s00210-010-0590-x

Wittwer, M.B., Zur, A.A., Khuri, N., Kido, Y., Kosaka, A., Zhang, X., et al. (2013). Discovery of potent, selective multidrug and toxin extrusion transporter 1 (MATE1, SLC47A1) inhibitors through prescription drug profiling and computational modeling. *J Med Chem* 56**,** 781-795. 10.1021/jm301302s

Yasujima, T., Ohta, K., Inoue, K., and Yuasa, H. (2011). Characterization of human OCT1-mediated transport of DAPI as a fluorescent probe substrate. *J Pharm Sci* 100**,** 4006-4012. 10.1002/jps.22548

Yonezawa, A., Masuda, S., Yokoo, S., Katsura, T., and Inui, K. (2006). Cisplatin and oxaliplatin, but not carboplatin and nedaplatin, are substrates for human organic cation transporters (SLC22A1-3 and multidrug and toxin extrusion family). *J Pharmacol Exp Ther* 319**,** 879-886. 10.1124/jpet.106.110346

Zhang, L., Dresser, M.J., Gray, A.T., Yost, S.C., Terashita, S., and Giacomini, K.M. (1997). Cloning and functional expression of a human liver organic cation transporter. *Mol Pharmacol* 51**,** 913-921. 10.1124/mol.51.6.913

Zhang, L., Gorset, W., Washington, C.B., Blaschke, T.F., Kroetz, D.L., and Giacomini, K.M. (2000). Interactions of HIV protease inhibitors with a human organic cation transporter in a mammalian expression system. *Drug Metab Dispos* 28**,** 329-334.

Zhang, L., Schaner, M.E., and Giacomini, K.M. (1998). Functional characterization of an organic cation transporter (hOCT1) in a transiently transfected human cell line (HeLa). *J Pharmacol Exp Ther* 286**,** 354-361.

Zheng, J., Chan, T., Zhu, L., Yan, X., Cao, Z., Wang, Y., et al. (2016). The inhibitory effects of camptothecin (CPT) and its derivatives on the substrate uptakes mediated by human solute carrier transporters (SLCs). *Xenobiotica* 46**,** 831-840. 10.3109/00498254.2015.1129080

Zhu, P., Ye, Z., Guo, D., Xiong, Z., Huang, S., Guo, J., et al. (2018). Irinotecan alters the disposition of morphine *via* inhibition of organic cation transporter 1 (OCT1) and 2 (OCT2). *Pharm Res* 35**,** 243. 10.1007/s11095-018-2526-y
